# *In Silico* Screening of the Key Cellular Remodeling Targets in Chronic Atrial Fibrillation

**DOI:** 10.1371/journal.pcbi.1003620

**Published:** 2014-05-22

**Authors:** Jussi T. Koivumäki, Gunnar Seemann, Mary M. Maleckar, Pasi Tavi

**Affiliations:** 1 Simula Research Laboratory, Center for Cardiological Innovation and Center for Biomedical Computing, Oslo, Norway; 2 Department of Biotechnology and Molecular Medicine, A.I. Virtanen Institute for Molecular Sciences, University of Eastern Finland, Kuopio, Finland; 3 Institute of Biomedical Engineering, Karlsruhe Institute of Technology, Karlsruhe, Germany; University of California San Diego, United States of America

## Abstract

Chronic atrial fibrillation (AF) is a complex disease with underlying changes in electrophysiology, calcium signaling and the structure of atrial myocytes. How these individual remodeling targets and their emergent interactions contribute to cell physiology in chronic AF is not well understood. To approach this problem, we performed *in silico* experiments in a computational model of the human atrial myocyte. The remodeled function of cellular components was based on a broad literature review of *in vitro* findings in chronic AF, and these were integrated into the model to define a cohort of virtual cells. Simulation results indicate that while the altered function of calcium and potassium ion channels alone causes a pronounced decrease in action potential duration, remodeling of intracellular calcium handling also has a substantial impact on the chronic AF phenotype. We additionally found that the reduction in amplitude of the calcium transient in chronic AF as compared to normal sinus rhythm is primarily due to the remodeling of calcium channel function, calcium handling and cellular geometry. Finally, we found that decreased electrical resistance of the membrane together with remodeled calcium handling synergistically decreased cellular excitability and the subsequent inducibility of repolarization abnormalities in the human atrial myocyte in chronic AF. We conclude that the presented results highlight the complexity of both intrinsic cellular interactions and emergent properties of human atrial myocytes in chronic AF. Therefore, reversing remodeling for a single remodeled component does little to restore the normal sinus rhythm phenotype. These findings may have important implications for developing novel therapeutic approaches for chronic AF.

## Author Summary

Atrial fibrillation is a complex disease which, at the level of individual atrial muscle cells, is a result of changes in a number of ion channels and transporters, as well as in cellular structure. How these alterations, together and separately, affect electrical and contractile function of the atrial cells is not well understood. In this study, we evaluated the effect of these changes using a computational approach. Our results show that abnormal function of both calcium and potassium ion channels at the sarcolemma has the largest impact on the electrical properties of the human atrial myocyte. Changes in intracellular calcium handling and cellular geometry are also significant for cellular function. Finally, our results highlight the interactions and additive effect of these abnormalities, in that a hypothetical restoration of any single modification does not result in recovery of function to a healthy phenotype. These findings have potentially important implications for developing novel treatment options for atrial fibrillation.

## Introduction

Atrial fibrillation (AF), the most common arrhythmia in clinical practice, is a complex disease with multiple etiologies [1]. However, the endpoint can be broadly characterized by two pathophysiological features: a tissue substrate with increased propensity to arrhythmia as well as loss of contractility. These global outcomes are due to adverse remodeling processes, leading to self-perpetuation of the arrhythmia [2], [3]. Despite its clinical significance the mechanisms of AF-induced contractile dysfunction are still poorly understood, and current drugs for the treatment of chronic AF (cAF) increase the risk of life-threatening arrhythmias while featuring only moderate efficacy [4].

In the literature, cAF-related remodeling is typically divided into three categories: (1) electrical, (2) contractile and (3) structural [5]. The first includes decreased conductances of L-type Ca^2+^ current (I_CaL_), transient outward K^+^ current (I_to_) and ultra rapid delayed rectified K^+^ current (I_Kur_), and increased conductance of inward rectified K^+^ current (I_K1_), and is considered a typical hallmark of cAF [6]. This electrical remodeling causes, for example, shortening of both the action potential (AP) duration and the effective refractory period (ERP). Contractile remodeling, on the other hand, appears to be predominantly a result of impaired intracellular Ca^2+^ handling, as contractile force can be almost completely restored by increasing the extracellular Ca^2+^ concentration [7]. Emerging evidence suggests that abnormal Ca^2+^ handling is a key contributor to atrial remodeling during AF [8]. The third category, structural remodeling, includes changes at both the cellular level (hypertrophy, glycogen accumulation and modified mitochondrial morphology, among others) and tissue level (fibrosis) [9].

It has been established in both *in vitro* and *in silico* experiments that the remodeling of sarcolemmal Ca^2+^ and K^+^ channels creates a substrate which supports the maintenance of AF [5]. Recent studies have also demonstrated that remodeled intracellular Ca^2+^ handling is one of the main causes for the loss of contractility observed in cAF [10], [11]. Furthermore, cellular hypertrophy has been shown to cause conduction disturbances, even in the absence of increased fibrosis [12]. However, neither how the above mechanisms interact nor how these may contribute as isolated modifications to alter the electrical and contractile function of atrial myocytes in cAF is well understood. To approach this complex problem, we conducted an extensive literature review to form a cohort of virtual cell variants that represent the various cellular components reported as remodeled in cAF. We then analyzed, both in single cells and in tissue, the mechanisms underlying AP shortening, altered intracellular Ca^2+^ signaling, and changes in excitability in cAF, using a recently developed mathematical model of the human atrial myocyte [13].

## Results

### Comparison of simulated results with experiments – model validation

**Figure 1:**
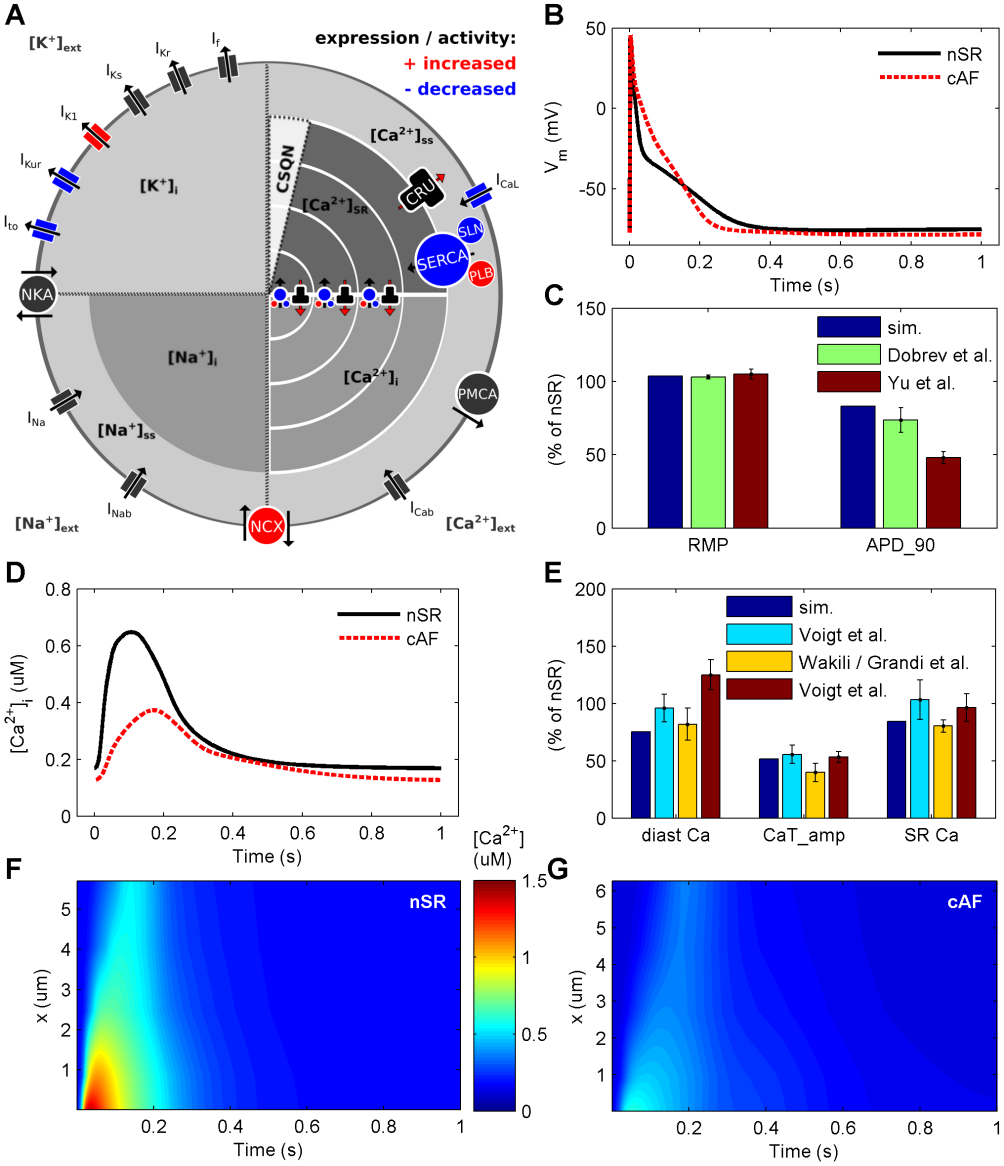
Illustration of cAF-remodeling processes accounted for in the model and consequent changes in electrophysiological properties and Ca^2+^ dynamics. (A) Schematic presentation of the cell model. Ionic currents and ion concentrations are referred to with I_X_ and [X^z^]_compartment_, respectively. Furthermore, NKA =  sodium potassium ATPase, NCX =  sodium Ca^2+^ exchanger, PMCA =  plasma membrane Ca^2+^ ATPase, SERCA =  sarcoplasmic reticulum Ca^2+^ ATPase, PLB =  phospholamban, SLN =  sarcolipin and CRU =  calcium release unit or ryanodine receptor. Colour coding with red and blue refers to increased and decreased activity and/or expression of cellular components (proteins involved in ion transport), respectively. (B & C) cAF-remodeling shortens the AP and hyperpolarizes the membrane. Simulation results are compared to *in vitro* findings of Yu et al. [42] and Dobrev et al. [43]. (D & E) cAF-remodeling decreases the amplitude of CaT, diastolic Ca^2+^ concentration and SR Ca^2+^ content, corresponding to *in vitro* results of Voigt et al. [21] (cyan bar), Voigt et al. [14] (red bar), Wakili et al. [10] (diastolic Ca^2+^) and Grandi et al. [11] (CaT_amp_ and SR Ca^2+^ content). (F & G) Spatiotemporal view of the CaT along the radial direction of the virtual cell in nSR and cAF (x =  distance from sarcolemma).

We initially compared the simulation results with *in vitro* findings for AP and CaT characteristics in cAF vs. normal sinus rhythm (nSR). The model reproduces one of the hallmarks of electrophysiological cAF-remodeling, AP shortening (Figure 1B). Measured as a decrease of APD_90_, in simulations AP shortening (31.9%) corresponds closely with previous *in vitro* studies (Figure 1C). In addition, the more negative (5.5% increase) resting membrane potential (RMP) observed here in cAF as compared to nSR cells is in line with experimental findings (Figure 1C).

Cellular cAF-remodeling also causes dramatic changes to Ca^2+^ dynamics (Figure 1D). In simulations, both reduced diastolic [Ca^2+^]_i_ (−29.1% for cAF vs. nSR) and the decreased CaT_amp_ (−62.3% for cAF vs. nSR) match *in vitro* findings well (Figure 1E). Additionally, a small reduction in sarcoplasmic reticulum (SR) Ca^2+^ content (−23% for cAF vs. nSR), measured as the integral of I_NCX_ during a caffeine pulse (Figure S2) is observed, which corresponds well with the 18% decrease reported *in vitro* in cAF myocytes [11]. Furthermore, CaT peak is delayed in simulations (by 49.8% for cAF vs. nSR), which compares well qualitatively with results obtained from a canine AF model [10]. The CaT decay time constant was also increased by 35.6% in cAF vs. nSR, within the reported range for *in vitro* results (28% [14] and 80% [11]). The spatiotemporal presentation of CaT in Figure 1 F&G shows there is virtually no rise in [Ca^2+^]_i_ in the central parts of the cAF-remodeled cell, which also corresponds well to *in vitro* findings [10].

Simulation results also accurately represent the non-linear nature of cardiac myocyte Ca^2+^ dynamics: although the maximum conductance of I_CaL_ is decreased by 65% in the cAF model as compared to the nSR model, the total Ca^2+^ influx is decreased only by 39.6% overall, as there is less Ca^2+^ dependent inactivation of I_CaL_ in cAF. These results are also in line with *in vitro* findings of 42% and 22% reduction in peak vs. integrated I_CaL_, respectively [14].

**Figure 2:**
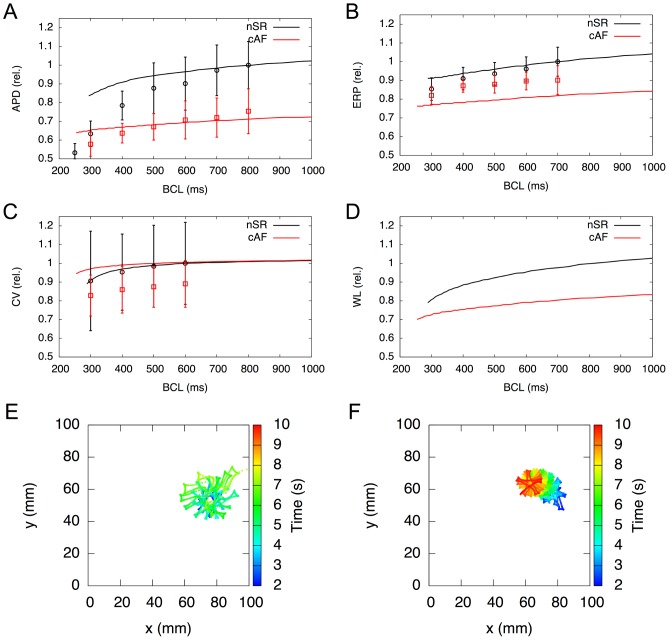
Electrophysiological properties in cAF tissue in silico. A–D) Restitution properties in a 1D tissue beam, compared to *in vivo* results of Franz et al. [27] for action potential duration at 90% repolarisation (A), Yu et al. [28] for effective refractory period (B), Feld et al. [29] for conduction velocity (C) and wavelength (D). Simulation results are normalised to BCL =  [0.8 0.7 0.6 1.0] s in (A–D), respectively. E&F) Mapping of rotor center trajectories after initiation in 2D tissue shows that in cAF (F) the meandering trajectory occupies a lot less space compared to nSR (E).

1D tissue simulations reveal restitution properties that also correspond well to *in vivo* findings in nSR vs. cAF (Figure 2 A–D). Relative APD_90_ and conduction velocity (CV) changes lie within the measured standard deviation. The model reproduces relative ERP for the nSR case quite well, although this is rather low for the cAF case. The rotor center in a 2D tissue patch for the nSR and the cAF *in silico* models are depicted in Figure 2 E and F, respectively. The rotor center trajectory of the nSR variant consumes greater area than that of cAF, representing the stabilization of reentrant waves associated with this electrophysiological remodeling. Furthermore, the rotor is meandering comparatively stable during the simulation time of 8 s for the cAF case, whereas the instable rotor center of the nSR case drifts collides with the geometry boundary and vanishes. Movies showing rotor movement for both cases can be found in Supporting Information (Video S1–S2). Simulated dominant frequencies are 4.15 Hz for the physiological case and 11.23 Hz for the cAF model, which compares favorably to the range of measured values of 11.6±2.9 Hz [15].

### Analysis of cellular remodeling targets in cAF

**Figure 3:**
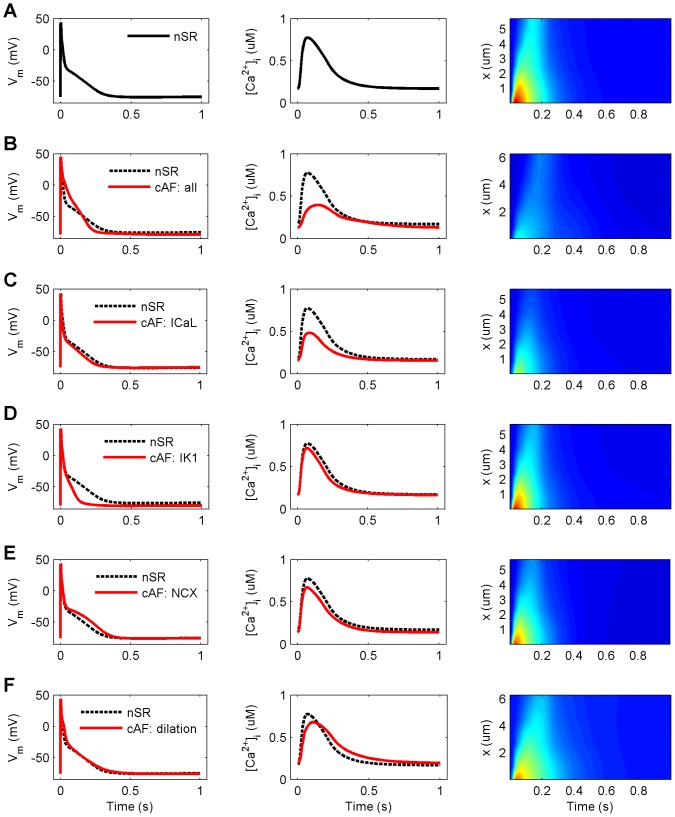
Effect of individual remodeling targets on CaT and AP characteristics in cAF. (A) normal sinus rhythm (nSR). (B) chronic atrial fibrillation (cAF: all). (C–F) four remodeled cellular components separately (L-type Ca^2+^ current, I_CaL_; inward rectified K^+^ current, I_K1_; Na^+^/Ca^2+^ exchanger current, I_NCX_; and increased cell volume, dilation), respectively. Columns from left to right: action potential (AP), Ca^2+^ transient (CaT) averaged over cell volume, and spatiotemporal presentation of CaT (x =  distance from sarcolemma). Colour scale for right column: 0–1.5 µM corresponds to dark blue – dark red (similar to Figure 1 F&G). All results are obtained at BCL = 1000 ms.

To evaluate the contribution of individual variables to cAF remodeling, we next simulated changes in each cellular component separately in the model and calculated three resultant biomarkers APD_90_, AP_tri_ and CaT_amp_ for all cell variants. Each cAF-remodeled component was included one at a time, and the AP and CaT characteristics of all cell variants were compared to the nSR myocyte (Figure 3). In simulations, reduced I_CaL_ alone decreased APD_90_ by 17.3%, while increased I_K1_ caused an even greater reduction of APD_90_ (by 52.7%). Increased I_NCX_ and cell dilation each had the opposite effect: APD_90_ increased by 21.5% and 7.4%, respectively. AP_tri_ was substantially decreased in cAF (−35.9%) vs. nSR, which appeared to be primarily related to increased I_K1_ and reduced I_CaL_, as these singular modifications reduced AP_tri_ by 58.9% and 16.6%, respectively. While cell dilation had almost no effect on AP_tri_ (+3.1%), cAF-remodeled NCX increased AP_tri_ quite dramatically (by 22.9%). On the other hand, CaT_amp_ was impacted most by reduced I_CaL_ (−46.0%), cell dilation (−20.0%) and increased NCX activity (−12.4%). These modifications also hampered the propagation of intracellular Ca^2+^ waves from sarcolemma to cell center (right column of Figure 3). The crucial role of increased I_K1_ and reduced I_CaL_ in cAF remodeling was further demonstrated in tachy pacing (BCL = 250 ms) simulations. Results showed that without these two remodeling targets, the virtual cell is unable to recover excitability between stimuli during such a fast pacing regime (Figure S5). Investigation of restitution properties in 1D tissue simulations (Figure S7) also revealed the dominant effects of changes in I_CaL_ and I_K1_ on APD, ERP, CV and WL restitution (as known from measurements and illustrated in Figure 2 A–C). Interestingly, the reduction of I_CaL_ alone led to alternans in electrical properties at higher rates.

Similar analysis using the same three biomarkers was also carried out for the cAF-remodeled I_to_, I_Kur_, SERCA and RyR; the results are shown in Supporting Information (Figure S3). Surprisingly, the effects of SERCA and RyR remodeling on both CaT_amp_ and AP morphology were very small as compared to the effects of, for example, cAF-remodeled I_CaL_ and NCX. However, it has been shown in animal studies that increased RyR sensitivity has only transient effects on CaT_amp_, as reduced SR Ca^2+^ content balances the effect of increased sensitivity [16]. Furthermore, increased PLB and decreased SLN expressions have opposing effects on the Ca^2+^ affinity of SERCA, so these modifications partially balance one another in the cAF model.

**Figure 4:**
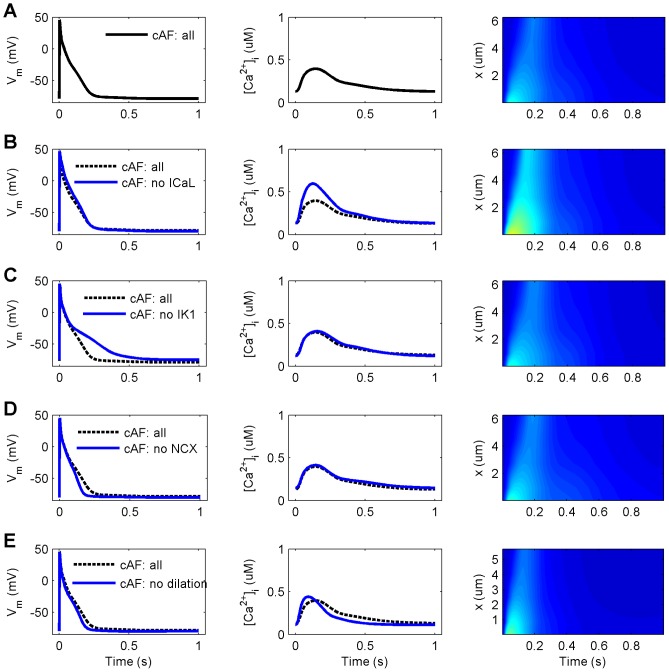
Effect of reversing remodeling of individual targets on CaT and AP characteristics in cAF. (A) chronic atrial fibrillation (cAF: all). (B–E) four restored cellular components (L-type Ca^2+^ current, I_CaL_; inward rectified K^+^ current, I_K1_; Na^+^/Ca^2+^ exchanger current, I_NCX_; and increased cell volume, dilation), respectively. Columns from left to right: action potential (AP), Ca^2+^ transient (CaT) averaged over cell volume, and spatiotemporal presentation of CaT (x =  distance from sarcolemma). Colour scale for right column: 0–1.5 µM corresponds to dark blue – dark red (similar to Figure 1 F&G). All results are obtained at BCL = 1000 ms.

To explore putative targets among the remodeled cellular components for reversing the cAF phenotype, we performed simulations in which we excluded each one of these components independently, and then compared AP and CaT characteristics to the full cAF model (Figure 4). Neglecting the effect of I_K1_ remodeling caused a substantial increase in APD_90_ (+79.7%), while similarly excluding the effects of I_CaL_ and NCX remodeling, as well as cell dilation had only relatively minor effects (+2.5%, −8.7% and +1.0% change in APD_90_, respectively). Interestingly, neglecting the effect of remodeled I_K1_ renders the virtual cell bistable: depending on initial conditions either a normal or unresponsive/depolarized steady-state is reached via normal pacing (BCL = 1000 ms; data not shown). The second biomarker, AP_tri_, was changed by −12.4%, 112.7%, −33.9% and −17.2% in comparison to cAF, when the remodeling of I_CaL_, I_K1_, NCX and cell dilation, respectively, were independently reversed. When compared to nSR, AP_tri_ values were not well restored: −43.8%, +36.4%, −57.6% and −46.9% for the I_CaL_, I_K1_, NCX and cell dilation, respectively. On the contrary, CaT_amp_ was almost completely restored when the effects of remodeling (reduced) I_CaL_ (+94.4%) were reversed, and enhanced to a smaller extent (+38.2%) if the virtual cell was not dilated (Figures 4 B&E, right column). The vital role of increased I_K1_ and reduced I_CaL_ in cAF remodeling was further demonstrated in tachy pacing (BCL = 250 ms) simulations; omitting either of these remodeling targets renders the virtual cell unresponsive to pacing at such a rapid rate (Figure S6). 1D restitution simulations revealed similar results as in single cell simulations (Figure S8). An increase of APD, ERP, CV and WL was only significant in the cases wherein I_CaL_ or I_K1_ remodeling were omitted. Interestingly, reduction of I_K1_ led to alternans at higher rates in this case. Similar analysis was performed for cAF-remodeled I_to_, I_Kur_, SERCA and RyR (results shown in Supporting Information, Figure S4).

### The strong link between intracellular Ca^2+^ and AP morphology persists in cAF

**Figure 5:**
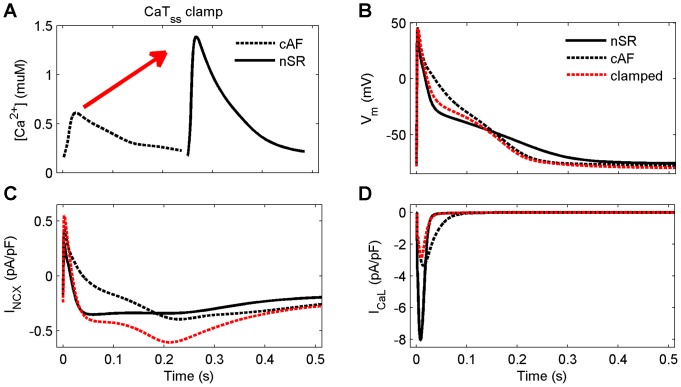
The strong link between intracellular Ca^2+^ and AP shape still exist in the cAF-remodeled virtual cell. Clamping the CaT (A) in the subsarcolemmal space to be normal (as in nSR) speeds up the initial and slows later repolarisation phases of membrane voltage (B), due to indirect changes in I_NCX_ (C) and I_CaL_ (D).

In a previous study, we showed that SR Ca^2+^ release is a strong modulator of APD [13]. Here, we used a similar approach to investigate to what extent AP shortening and triangulation in cAF might be reversed if intracellular Ca^2+^ dynamics were restored to match those in nSR. Figure 5A shows the subsarcolemmal CaT (CaT_ss_) clamp used in simulations. Restoring CaT_ss_ had substantial effect on AP shape, increasing APD_90_ by 18.9% and AP_tri_ by 16.1%. Figures 5 C and D illustrate the underlying changes in I_NCX_ and I_CaL_ responsible for modifying these late and early stages of repolarization, respectively.

### Na^+^ accumulation during fast pacing

**Figure 6:**
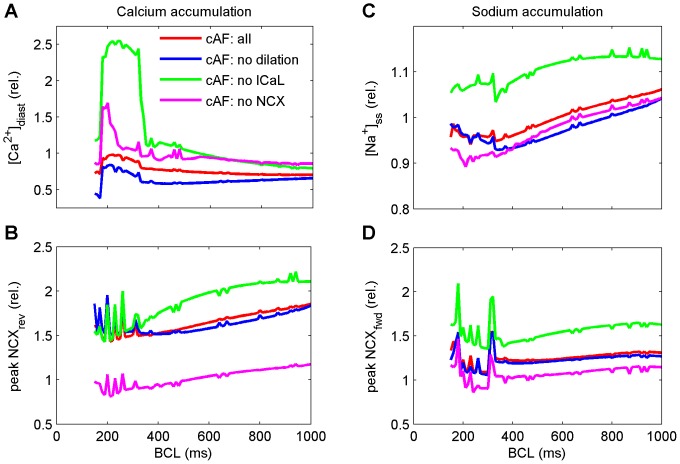
Intracellular Ca^2+^ and Na^+^ accumulation in five virtual cell variants during increasingly fast pacing. (A&B) Ca^2+^ accumulation and corresponding activation of the reverse mode of NCX in four cell model variants (normalized to nSR). (C&D) Na^+^ accumulation and corresponding activation of the forward mode of NCX (normalized to nSR).

As the typical rate of electrical activation of cells in cAF is dramatically faster than in nSR, we next analyzed accumulation of intracellular Na^+^ and Ca^2+^ during increasingly fast pacing (Figure 6 B and C). Previous studies have already established that intracellular Na^+^ accumulation, which is inherently linked to Ca^2+^ accumulation via NCX, is an important mechanism for AP shortening during fast pacing [11], [13]. Motivated by the finding that restoring the intracellular CaT appears to impart a beneficial effect on AP shape (increased APD_90_; Figure 5), we analyzed the effect of independently reversing remodeling of cellular components affecting CaT properties the most: I_CaL_ reduction, increased NCX, and cell dilation. As results in Figure 6 reveal, reversing remodeling of I_CaL_ affects Na^+^ and Ca^2+^ accumulation most dramatically of the three. Interestingly, in addition to increasing the magnitude of ion accumulation, there is also dramatic shift in the ionic dynamics. Specifically, when I_CaL_ is restored to a “healthy level” in a cAF-remodeled virtual cell, the regime of Ca^2+^ overload is shifted to larger, more physiologically relevant BCLs (Figure 6A). Similar analysis was performed for all the other cell model variants (results shown in Supporting Information, Figure S10).

To show directly that Na^+^ accumulation is still a mechanism responsible for AP shortening in drastically remodeled cells, we clamped Na^+^ concentration to its steady-state value when pacing the model at BCL = 1000 ms, while all other variables represent a steady-state at BCL = 167 ms. The late phase of AP repolarization is slowed substantially (Figure S9E) during Na^+^ clamp, as there is less intracellular [Na^+^] to activate the Na^+^/K^+^ ATPase (NKA) current (Figure S9F) than when Na^+^ is allowed to accumulate normally.

### Inducibility of DADs in cAF-remodeled cells

**Figure 7:**
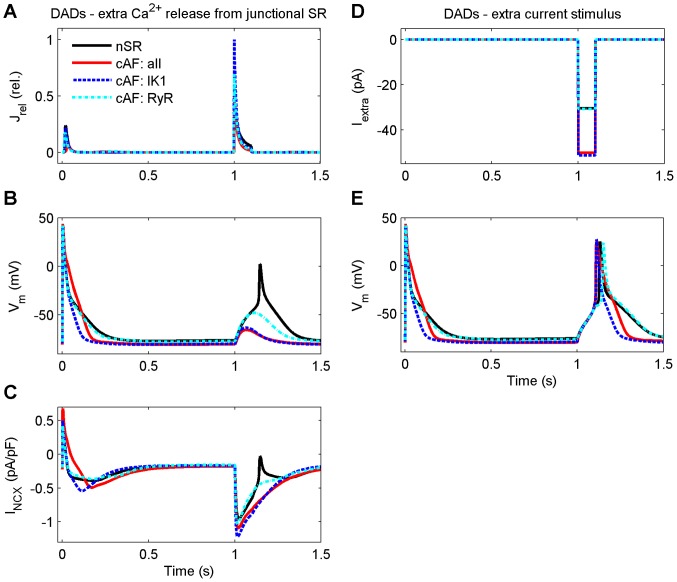
Inducibility of DADs is decreased in cAF due to the stabilizing effect of remodeled I_K1_. DADs were induced with either an extra Ca^2+^ release from the junctional sarcoplasmic reticulum (A–C) or an extra current stimulus (D&E) at the time point of 1 second. Same protocol was used to study four model variants: normal sinus rhythm (nSR), chronic atrial fibrillation with all modifications (cAF: all), only modified inward rectified K^+^ current (cAF: IK1), and only ryanodine receptor Ca^2+^ sensitivity (cAF: RyR).

Delayed afterdepolarizations (DADs) have been linked to various arrhythmogenic diseases; however their role in cAF has not yet been elucidated [17]. The main mechanism for induction of cellular DADs in human atrial cells has been shown to be NCX [18]. It was thus of special interest to examine, how remodeled Ca^2+^ handling might affect the inducibility of DADs in these cells. First, we tested whether DADs could be induced via an extra opening of RyRs during diastole (Figure 7A). While it was not possible to induce DADs in the cAF-remodeled virtual cell (Figure 7B), the subsequent activation of NCX (Figure 7B) in the nSR model variant did elicit DADs, as the inward current sufficiently depolarized the virtual cell to elicit an AP. A possible explanation for this surprising finding is the reduced SR Ca^2+^ content in cAF cells. To test this, we employed the same protocol used in cAF versus nSR virtual cells in a cell variant featuring RyR remodeling only (with all other features identical to nSR model). Even maximal opening of the RyR was not enough to activate NCX and induce a DAD in this variant (*cAF: RyR* in Figure 7), supporting the hypothesis that it was not possible to induce DADs in cAF cells due to reduced SR Ca^2+^ content. In this case, however, membrane potential following RyR opening was closer to the AP initiation threshold than in the original cAF cell variant (Figure 7B).

An additional mechanism to explain the lack of DADs in the cAF-remodeled cell could be increased I_K1_, which might stabilize the membrane potential such that pathological opening of RyRs during diastole would not induce a DAD. To investigate this possibility, we performed simulations in which DADs were induced by current injection during diastole (Figure 7D). A 65% greater current amplitude was needed to induce a DAD in cAF as compared to nSR, suggesting that the cAF model membrane was indeed more stable with respect to depolarization. To further isolate the role of increased I_K1_, we ran corresponding simulations in a model variant that included only the remodeling of I_K1_ (*cAF: IK1* in Figure 7). Compared to nSR, a 69% greater current amplitude was needed to induce a DAD in this model variant (Figure 7D).

These results implicate two mechanisms that dramatically reduce the inducibility of DADs in cAF-remodeled virtual cells. First, when SR Ca^2+^ content is reduced, it is not possible to release a sufficient amount of Ca^2+^ from the SR to activate NCX to the extent that would elicit a DAD. Second, increased I_K1_ decreases cell excitability and stabilizes the membrane potential against DADs in cAF, as with a RMP hyperpolarization of −3.7 mV, a larger depolarizing current is needed to reach the AP threshold. Thus, reduced SR Ca^2+^ load together with increased I_K1_ actually overcompensates for the combined, contradictory effects of increased RyR sensitivity and increased expression of NCX, such that DAD inducibility is reduced rather than enhanced in our *in silico* model of cAF.

### Central targets in cAF remodeling

**Figure 8:**
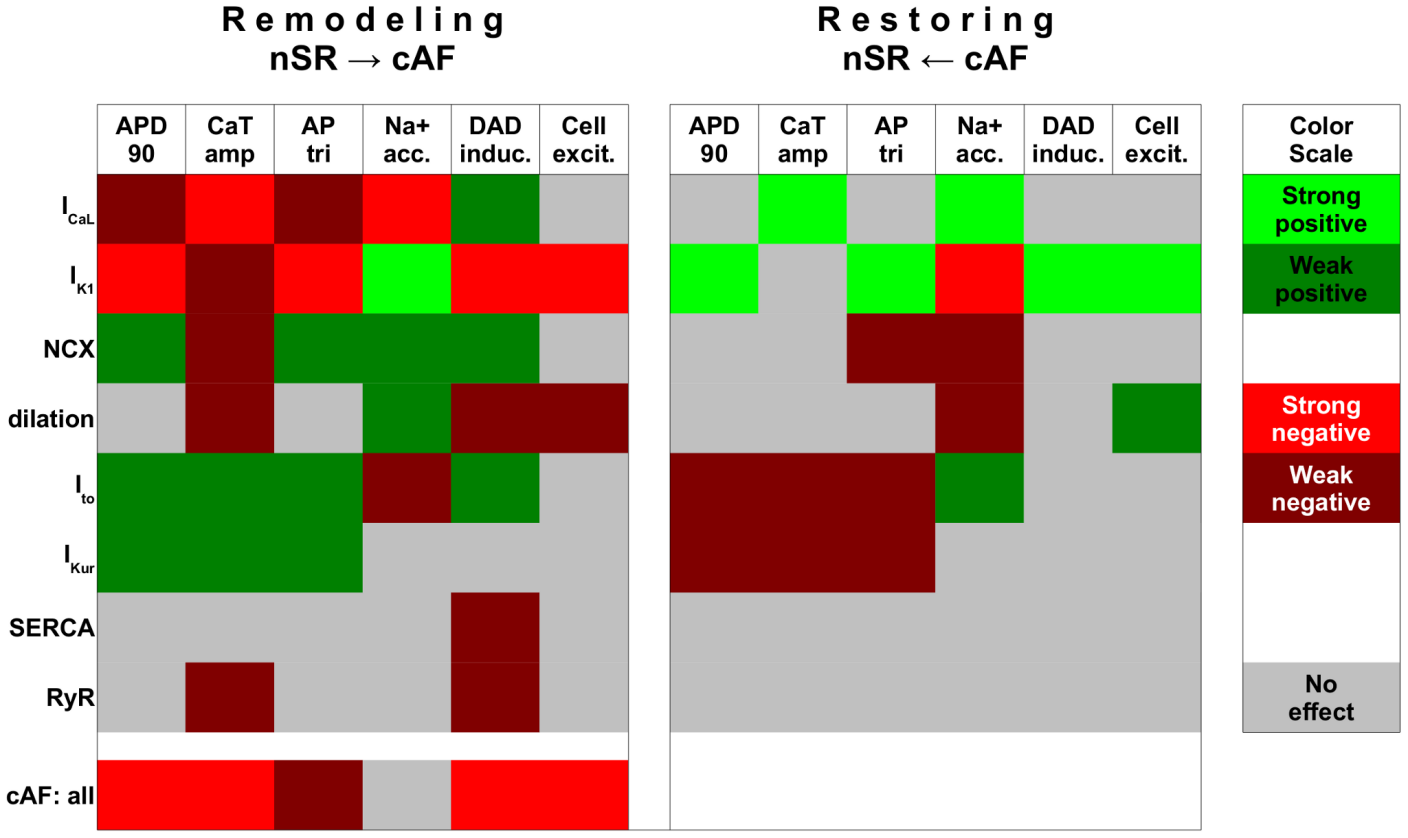
Summary of cellular remodeling affecting electrophysiological properties in cAF. Analysis was done for both individual inclusion (Remodeling) and exclusion (Restoring) of cellular components to elucidate their contribution action potential duration at 90% repolarisation (APD_90_), Ca^2+^ transient amplitude (CaT_amp_), action potential triangulation (AP_tri_), Na^+^ accumulation, inducibility of delayed afterdepolarisations (DAD induc.) and electrical excitability of the cell (cell exc.).

Simulation results are summarized in Figure 8 in a heat map-like presentation, where each individual modification is rated as based on its impact in remodeling (from nSR to cAF) and in reverse remodeling (from cAF closer to nSR) on selected AF biomarkers. It is apparent that individual modifications have differential impact on cell function depending whether each is involved in remodeling or reverse remodeling (i.e. the isolated modification is aimed at improving function following cAF remodeling). For example, while cell dilation affects several functional variables (CaT, Na^+^ accumulation, DAD inducibility and cell excitability) during the remodeling process, when excluded from cAF-remodeled cells, cell dilatation affects only cell excitability. It is also clear that the cAF phenotype is more resistant to modifications than the nSR phenotype (see larger gray area in Figure 8). In evaluating the impact of isolated features of remodeling, results also show that the increase in I_K1_ is the most central component in cAF remodeling, since it affects all functional markers.

## Discussion

### Ca^2+^ signaling is altered in cAF

The importance of abnormal intracellular Ca^2+^ handling in the pathophysiology of AF is becoming clear [19], [20]. According to our simulations, Ca^2+^ signals are severely blunted in cAF (smaller CaT_amp_), which corresponds well with experimental data [10], [11], [14], [21]. These changes are primarily due to decreased I_CaL_, and secondarily to the increased activity of NCX. Changes in other remodeling targets involved in Ca^2+^ handling, such as RyR and SERCA, had only minor impact on Ca^2+^ signals (Figures 3 and 4 and Figures S2 and S3). Reduction in I_CaL_ exerts its effect not only by limiting the Ca^2+^ influx and thus the immediate trigger for Ca^2+^-induced Ca^2+^ release during the AP, but also reduces SR Ca^2+^ content over time, thereby further reducing the strength of SR Ca^2+^ release.

In atrial myocytes, Ca^2+^-induced Ca^2+^ release involves two separate phases: the Ca^2+^ influx first activates RyR clusters in the vicinity of the sarcolemma and Ca^2+^ release from these junctional release sites triggers a propagating Ca^2+^ wave activating adjacent RyRs located deeper within the cell [10], [13], [22]. However, recent data suggest that larger mammals, including humans, might actually have a more developed network of t-tubules in their atrial cells than previously thought [23]. In fact, t-tubules are present in the ovine atrial myocytes at low density and strongly reduced in AF, leading to gradual loss of synchronization of Ca^2+^ signals [24]. This feature of cAF-remodeling is a very interesting topic for future study, particularly as increased spatial heterogeneity in Ca^2+^ diffusion within the cell has been shown to promote the genesis of Ca^2+^ alternans [25].

Our simulations and *in vitro* data [10] have shown the vulnerability of the fire-diffuse-fire mechanism to disruption in cAF; suppression of Ca^2+^ influx during AF remodeling leads to severely compromised transverse propagation of Ca^2+^ inside the cell. Ca^2+^ ions are thus only circulated within the volume just beneath the sarcolemma in this case; a defect aggravated by cAF-induced cell dilatation, which increases the volume of the junctional space and further dilutes [Ca^2+^] in the subsarcolemmal space. This redistribution of Ca^2+^ is likely to contribute to the suppression of contraction during AF, as with impaired propagation, Ca^2+^ signals do not reach the contractile elements located centrally within the cell. Furthermore, this alteration likely has a profound indirect effect on energy expenditure of the cAF-remodeled cells, since contractile elements are not activated to same extent and thus consume less energy than in healthy cardiomyocytes [26].

According to experimental data from patients suffering from chronic AF, contractile force of atrial tissue can be restored with increased extracellular [Ca^2+^] [7]. This implies that remodeling of the cellular contractile elements involved in cAF has a lesser role in depressing contraction in cAF as compared to the impact of altered Ca^2+^ signaling in the disease. Furthermore, the data also suggest that Ca^2+^ influx is the single most influential variable when considering cAF-induced contractile dysfunction in the light of electrical remodeling. This view is supported by our simulations, wherein cAF-induced I_CaL_ downregulation alone reduces CaT_amp_ by 46% and induces defects in Ca^2+^ signal propagation (Figure 3).

### AP morphology changes in cAF

A number of remodeling targets in AF have been proposed to contribute to changes in the AP waveform [5], [17]. Our simulations support the conclusion that the two most important elements leading to AP shortening in AF are increased I_K1_ and decreased I_CaL_. Increased I_K1_ alone reduces APD by 52.7% and is also the single most influential factor contributing to AP triangulation (Figure 3). Furthermore, the membrane potential is hyperpolarized in diastole by the cAF-related remodeling of I_K1_ (which is a repolarizing current and one of the main contributors to the maintenance of the RMP in cardiac myocytes). Interestingly, some of the remodeling modifications also act to lengthen the AP, e.g. increases in I_NCX_ and cell volume. Increase in the NCX current promotes augmented inward current during AP repolarization when it exchanges cytosolic Ca^2+^ ions for extracellular Na^+^ ions at a ratio of 1∶3. The effect of the cell volume on AP is a bit more indirect; the volume increase appears to delay the Ca^2+^ removal from the cytosol, which in turn increases I_NCX_ during late repolarization (Figure 3).

Intracellular Ca^2+^ signals and the AP are tightly coupled in human atrial myocytes inherently, and this coupling seems to be an essential part of AF remodeling. Hence, as compared to changes in e.g. K^+^ current densities which have more straightforward effects on AP, changes in variables involved in Ca^2+^ signaling, like I_CaL_ and I_NCX_, modulate not only the AP directly, but have more adverse consequences through their effects on intracellular Ca^2+^ signals. In our simulations, remodeling of I_CaL_ alone reduced APD_90_ by 17.3% (compared to AP shortening by 31.9% in cAF, Figure 3), while normalization of CaT in cAF cells lengthened the AP by 18.7% (Figure 5). This suggests that effects of I_CaL_ on AP are mediated only partly by direct impact of the current on V_m_ and that major effects come via the secondary suppression of CaT.

### Tissue electrophysiology is influenced by cAF

Changes in AP morphology also impact tissue electrophysiology (see Figure 2). The simulated tissue APD_90_ is reduced by around 30%, which is in agreement with the available *in vivo* data [27]. Similarly, the simulated ERP is reduced by about 20%. In this case, the measured data from Yu et al. [28] revealed reduction by a lesser extent (around 10%) as compared to simulation data. These differences might be due to different stages of remodeling. CV is not influenced to a great degree in our simulations, as we did not include gap junction remodeling. Feld et al. [29] measured a reduced CV in cAF, suggesting that there might be changes in conductive tissue properties during cAF. The reduced wavelength (the product of ERP and CV) in the simulated cAF case suggests the higher chance of the maintenance of AF following rotor initiation. The simulated rotor center trajectories (Figure 2) show that these anchor more easily in cAF, evincing greater stability, whereas in nSR, the rotor core tends to meander and subsequently might be eliminated at a boundary or an anatomical obstacle. The simulated dominant frequencies also demonstrate the higher chance of a permanent fibrillation in the cAF case.

### Sodium accumulation is attenuated in cAF

Intracellular Na^+^ accumulation has been established as an important mechanism for AP shortening during fast pacing in previous studies [11], [13]. In cardiac myocytes, [Na^+^]_i_ is mainly dictated by the balance between Na^+^ influx during an AP (upon activation of I_Na_ and I_NCX_) and Na^+^ efflux (through NKA and NCX). Therefore, Na^+^ fluxes are tightly coupled with both [Ca^2+^]_i_ and activation frequency, both of which are drastically altered in AF. According to our simulations, high frequency activity induces substantial Na^+^ accumulation in cAF cells, and this accumulation acts to shorten the AP upon activation of NKA, although this mechanism is less prominent than in nSR cells. In all types of cardiac myocytes, Na^+^ accumulation can result indirectly via Ca^2+^ overload which itself automatically results from high frequency pacing. In cAF cells, Ca^2+^ overload is limited by remodeling (reduced I_CaL_), which drastically suppresses the AP-evoked CaT. Thus, there is less Ca^2+^ to be extruded by NCX and consequently a lesser degree of Na^+^ accumulation.

To demonstrate the link between [Ca^2+^]_i_ and [Na^+^]_i_, we normalized the I_CaL_ in cAF model and noticed that pacing-induced Ca^2+^ and Na^+^ accumulation were both augmented (Figure 6). It could be hypothesized that altered Ca^2+^ and Na^+^ balances are actually among the features of cAF cells that enable sustained high frequency activity. When cardiomyocytes act to restore normal levels of [Ca^2+^]_i_ and [Na^+^]_i_, vast amounts of ATP are consumed by SERCA (to pump Ca^2+^ to the SR) and by NKA (to pump Na^+^ to the extracellular space). Thus, when ion gradients are smaller, cAF cells can maintain high frequency of activation at lower energy costs [26].

### Inducibility for DADs is not increased in cAF-remodeled cells

The main mechanism for induction of cellular DADs in human atrial cells has been shown to be activation of NCX [18]. As NCX is overexpressed in cAF, we expected to see a lower threshold for DADs in simulations; however, results revealed the opposite finding. In fact, an extra Ca^2+^ release from the SR was not enough to trigger DADs in the cAF-remodeled virtual cell. This result appears to contradict the recent *in vitro* findings that showed increased spontaneous Ca^2+^ waves in cells of AF patients [30], [31]. Possible explanations for this discrepancy include the measurement conditions (experiments carried out at room temperature) and pooled patient population (no separation for paroxysmal, persistent and chronic AF). Indeed, further analysis of our simulation results showed that the mechanism explaining this surprising finding was the reduced cellular excitability due to increase of inward rectifying K^+^ currents.

Our results suggesting reduced DAD inducibility in cAF contradict the recent finding that enhanced SR Ca^2+^ leak and NCX function underlie DADs in patients with cAF [14]. In another study, however, DADs were not observed in either nSR nor cAF patient tissue despite the fact that the measurement conditions were in favor of such events, as I_Kur_ was blocked with AVE0118 compound [32]. These controversial results suggest that increased propensity for DADs in cAF might depend, for example, on underlying etiologies in the patient population.

To summarize, DAD inducibility depends on four factors mechanistically: 1) the strength of the input (SR Ca^2+^ load), 2) how this input is transformed into a trigger (sensitivity of RyR), 3) how much depolarizing current this trigger induces (NCX vs. SERCA balance in Ca^2+^ removal from the cytosol), and, ultimately, 4) if the depolarizing current is large enough to depolarize the membrane voltage above the threshold for I_Na_ activation (which depends on the dynamic balance of depolarizing and repolarizing membrane currents). All four factors are altered in the context of cAF. Because factors #2 and #3 are greater (increase RyR sensitivity and greater net depolarizing current, respectively) one might intuitively infer that DAD inducibility would be increased in cAF-remodeled cells. In our *in silico* cAF model, however, the reduced SR Ca^2+^ load together with increased I_K1_, which reduce the trigger and stabilize the resting membrane potential, respectively, overcompensate for the combined depolarizing effect of increased RyR sensitivity and increased expression of NCX, such that DAD inducibility is actually reduced.

Future computational studies, possibly employing stochastic methods and finer spatial resolution, should address factors #2 and #3 in more detail, when *in vitro* data on the co-localization of RyR and NCX in cAF vs. nSR human atrial cells becomes available.

### What are the key cellular components contributing to remodeling in cAF?

Both AP shortening and loss of contractility are hallmarks of cAF. Our analysis indicates that, at the cellular level, these changes are strongly coupled to the increased I_K1_ and decreased I_CaL_ conductances, respectively. In fact, without the I_K1_ modification, the cAF-remodeled cell becomes unresponsive during more rapid pacing due to sustained depolarization of the membrane voltage (which inactivates fast sodium channels). Decreased I_CaL_ conductance, on the other hand, has a more diverse effect. While also contributing to AP shortening, reduced I_CaL_ is the main mechanism for the diminished intracellular CaT_amp_ in cAF. The large impact of the remodeling of I_CaL_ is related to its dual role, since it not only acts as a trigger for Ca^2+^ release from the SR, but also affects Ca^2+^ loading of the SR.

Remodeled I_CaL_ and I_NCX_ work in synergy to adapt the cell to abnormally fast reoccurring activation in cAF. While reduced I_CaL_ and increased I_NCX_ both reduce Ca^2+^ overload during fast pacing, they also shift Ca^2+^ dynamics from the normal “whole-cell state” to a “subsarcolemmal state”, where Ca^2+^ cycling is limited primarily to the vicinity of the cell membrane. Myocyte hypertrophy exacerbates the effect of remodeled Ca^2+^ handling, in that it further reduces CaT_amp_ in cAF in addition to the effects of I_CaL_ and NCX remodeling. The dilation of the cell also increases the delay between the peaks of the AP and the CaT, which may have arrhythmogenic effects in tissue. In fact, Schotten et al. [12] found that myocyte hypertrophy can cause conduction disturbances in the absence of increased fibrosis in a goat model of chronic atrio-ventricular block. As changes in intracellular Ca^2+^ signaling are centrally involved in normal and pathological regulation of myocyte growth, apoptosis and necrosis [33], cell dilation warrants further research to elucidate its role in cAF.

### What could be the key targets for restoring cell function in cAF?

Anti-arrhythmic drug therapy to counter AF has long concentrated on agents that may delay atrial repolarization. Drug targets have included, for example, I_Kr_ and I_Ks_; however, more recently agents blocking I_Kur_ have been studied extensively, because of the current's atria-specificity in human myocardium. More recently, intracellular Ca^2+^ handling has been established as a potential drug target in cAF [19]. As our results showed (Figure 5), restoration of intracellular CaT could, hypothetically, be used to improve AP shape (increase APD_90_) in cAF to, for example, lengthen the effective refractory period.

The most effective targets for restoring healthy cell properties following cAF-induced electrical remodeling are likely to be those that most impact the cAF phenotype. Our simulations suggest that changes in I_K1_ and I_CaL_ in isolation induce most of the characteristic features of cAF (Figure 8). Therefore, restoring either the K^+^ or Ca^2+^ conductance could, in theory, be effective in limiting the effects of electrical remodeling in the cAF substrate. However, complete reversal of any single cAF-induced change via pharmacological means is not likely to be feasible. Instead, it might be useful to consider therapies that aim at partial restoration of combinations of targets. In such efforts, however, understanding the full implications of altered cellular electrophysiology on tissue and organ dynamics is absolutely essential. To illustrate, consider the partial inhibition of K^+^ currents (for increasing APD_90_ and thus ERP), in combination with drugs aimed at increasing CaT_amp_ (for restoring contractility). Partial block of NKA with digoxin, combined with reduced RyR Ca^2+^ leak using a calmodulin kinase II inhibitor, appeared to be beneficial in single cell simulations (Figure S11) and may actually become feasible in the near future, as novel specific blockers of I_K1_ are being developed [34]. The 1D restitution results (Figure S12) also illustrated increases in APD, ERP, CV and WL, which may be desirable in terms of protecting against arrhythmia. However, this model variant developed alternans at higher pacing rates. In 2D simulations, these alternans also led to a break-up of a single rotor into two rotors (not captured within the geometry, so excitation vanishes; Figure S12E and Video S3). In a realistic geometry, such wavebreak could lead to stable fibrillatory activity. This finding highlights the need to carry out *in silico* analysis of potential drug targets at different scales (cell, tissue, organ) to achieve a more realistic understanding of pharmacological effects.

### Limitations of the study

Although the human atrial myocyte model employed here has been shown to be the most internally consistent and physiologically accurate to date, particularly regarding intracellular Ca^2+^ handling, in a recent comparison, the model has its limitations [35]. Furthermore, a holistic analysis of cAF as effected in this study is inevitably biased to some extent by the fact that the pathophysiology clinically involves multiple etiologies. Some studies group available data based on, for example, whether patients have a valvular disease or not, while other studies pool the data among AF subtypes and etiologies. Finally, our model of the cAF-remodeled cell is by no means exhaustive, as novel mechanisms of electrical remodeling are reported continuously. Instead, we have included those remodeling targets that have been established in more than one study of human atrial electrophysiology. When novel experimental data on these disease mechanisms accumulate, the model should be updated accordingly.

### Conclusions

The results indicate that, at the cellular level, reduced I_CaL_ and increased I_NCX_ contribute synergistically to adapt the cell to fast activation rates of cAF by reducing Ca^2+^ overload, which additionally causes a drastic decrease in CaT_amp_ at normal heart rates. Furthermore, our findings suggest that an increase of I_K1_ in cAF is the dominant mechanism responsible for AP shortening in cAF, while the effect of reduced I_CaL_ is less prominent and the role of remodeled I_to_ and I_Kur_ are rather insignificant. Increased I_K1_, in synergy with reduced intracellular Ca^2+^ stores, also stabilizing the cAF-remodeled cell against DADs. The results also show that, in addition to remodeling of ion currents and Ca^2+^ handling, cellular hypertrophy is an important mechanism contributing to changes in atrial refractoriness, contractility and arrhythmogenicity. Finally, the intrinsic complexity and interdependency of electrophysiological mechanisms are highlighted by our analysis. The presented results thus suggest that instead of targeting a single cellular component a more holistic approach is worth considering when looking for novel therapeutic approaches for chronic AF.

## Materials and Methods

The modeling platform of this study is our recently developed human atrial myocyte model that enables the simulation of emergent spatiotemporal characteristics of intracellular Ca^2+^ dynamics [13]. Methods for simulation of tissue-level electrophysiology and its analysis are presented in the Supporting Information and are detailed in [35]. Contrary to most previous *in silico* studies of cAF, we performed a broad literature search on cellular remodeling to define the average remodeled parameter values (Figure 1A) instead of using a single *in vitro* data set or small subset. We have included those remodeling targets that have been established in more than one study. Full sets of referenced human data are shown in Supporting Information (Tables S2–S4).

The modifications of existing model components, as well as the simulation protocols are described in detail in the Supporting Information. Briefly, we reformulated the I_CaL_ to increase the contribution Ca^2+^-dependent vs. voltage-dependent inactivation of the current, and decreased the time constants based on recent *in vitro* data [36], Supporting Information Figure S1. Parameters of the SERCA pump have been modified according to a previously developed scheme [37], [38] to enable the representation of changed expression of phospholamban (PLB) and sarcolipin (SLN) in cAF.

In our analysis of cAF-related cellular remodeling, we use the following three biomarkers:

1. APD_90_: AP duration at 90% repolarization. Because atrial refractoriness depends on APD, AP shortening effectively increases vulnerability of the tissue as a substrate for AF [3].
2. AP_tri_: AP shape during the later and final parts of the repolarization, calculated as the difference between APD_50_ and APD_90_ (AP_tri_  =  APD_90_–APD_50_). Triangulation of AP has been shown to be pro-arrhythmic [39], and is considered as one of the hallmarks of AF [40].
3. CaT_amp_: amplitude of the intracellular Ca^2+^ transient; difference of systolic and diastolic intracellular Ca^2+^ concentrations ([Ca^2+^]_i_). Reduced CaT_amp_ leads to loss of contractility at the tissue level, increasing the risk of stroke and thrombosis in cAF [41].

## Supporting Information

Figure S1 Characteristics of the modified I_CaL_ submodel. (A) Modified time constants of inactivation and recovery, based on in vitro data of Li *et al*. [44] and Christ *et al*. [36]. (B) Modified Ca^2+^-dependent inactivation gate. (C) Results of an in silico voltage clamp experiment, with 10 mM EGTA. (D) Voltage clamp protocol: BCL = 5000 ms, holding potential −80 mV, 1500 ms ramp to −40 mV to inactivate I_Na_, and pulse length of 300 ms at each testing potential.
(TIF)

Figure S2 Simulated caffeine experiment. In cAF, the amplitude of the caffeine-evoked CaT (A) is decreased, in line with the *in vitro* results (D) of Voigt et al. [21] (cyan bar), Grandi et al. [11] and Voigt et al. [14] (yellow bar), while the integral of I_NCX_ is affected to a much smaller extent (B & D). (C & E) Spatio-temporal properties of caffeine-evoked CaT are also changed due to the cAF-remodeling of Ca^2+^ handling.
(TIF)

Figure S3 Contribution of each remodeled cellular component to changes in AP and CaT characteristics. (A) normal sinus rhythm (nSR). (B–E) four remodeled cellular components separately (I_Kur_, I_to_, RyR, SERCA), respectively. Colour scale for right column is same as in Figure 3&4 0–1.5 µM corresponds to dark blue – dark red.
(TIF)

Figure S4 AP and CaT characteristics when a single remodeled cellular component is omitted. (A) chronic atrial fibrillation (cAF: all). (B–E) four restored cellular components (I_Kur_, I_to_, RyR, SERCA), respectively. Colour scale for right column is same as in Figure 3&4; 0–1.5 µM corresponds to dark blue – dark red.
(TIF)

Figure S5 Contribution of each remodeled cellular component to changes in AP and CaT characteristics during tachy pacing (BCL = 250 ms, red solid line) as compared to normal pacing (BCL = 1000 ms, blue dashed line). (A) normal sinus rhythm (nSR) model at slower pacing (BCL = 500 ms). (B) normal sinus rhythm (nSR) model. (C) chronic atrial fibrillation (cAF: all) model. (D–G) four remodeled cellular components separately (L-type Ca^2+^ current, I_CaL_; inward rectified K^+^ current, I_K1_; Na^+^/Ca^2+^ exchanger current, I_NCX_; and increased cell volume, dilation), respectively. Colour scale for right column is same as in Figure 3&4; 0–1.5 µM corresponds to dark blue – dark red. Model variants are identical to Figure 3.
(TIF)

Figure S6 AP and CaT characteristics when a single remodeled cellular component is omitted during tachy pacing (BCL = 250 ms, red solid line) compared to normal pacing (BCL = 1000 ms, blue dashed line). (A) chronic atrial fibrillation (cAF: all). (B–E) four restored cellular components (L-type Ca^2+^ current, I_CaL_; inward rectified K^+^ current, I_K1_; Na^+^/Ca^2+^ exchanger current, I_NCX_; and increased cell volume, dilation), respectively. Colour scale for right column is same as in Figure 3&4; 0–1.5 µM corresponds to dark blue – dark red. Model variants are identical to Figure 4.
(TIF)

Figure S7 Contribution of each remodeled cellular component to normal electrophysiological properties in 1D tissue simulation. (A) APD (B) ERP, (C) CV and (D) WL.
(TIFF)

Figure S8 Contribution of omitting each remodeled cellular component to chronic AF electrophysiological properties in 1D tissue simulation. (A) APD (B) ERP, (C) CV and (D) WL.
(TIFF)

Figure S9 Intracellular Ca^2+^ and Na^+^ accumulation during increasingly fast pacing. (A–D) Raw data (corresponding to Figure 6 B–E), and (E–F) effect of Na+ clamp on AP and INKA. Na^+^ clamp, at BCL = 167 ms, was simulated by setting intracellular Na^+^ concentration to the value at BCL = 1000 ms.
(TIF)

Figure S10 Intracellular Na^+^ accumulation during increasingly fast pacing for all the model variants, either including (A) or excluding (B) only a single remodeling target.
(TIF)

Figure S11 Restored RyR Ca^2+^ sensitivity and I_K1_ conductance with blocking of Na^+^/K^+^ ATPase (NKA) as an approach to increasing APD and CaTamp. (A) Faster initial and slower final phase repolarization. (B) Increased amplitude and peak CaT. (C&D) Indirect effects on I_CaL_ and I_NCX_. Partial block of NKA was implemented by reducing maximum current/conductance by 25%. RyR leak reduced by using the Ca^2+^ sensitivity and I_K1_ conductance, i.e. these properties were set similar to nSR.
(TIF)

Figure S12 Restitution properties of the restored model variant in comparison with nSR and cAF. (A) APD (B) ERP, (C) CV and (D) WL. The restoring increases the WL almost to the level of nSR but alternans are visible for higher rates. (E) The rotor trajectory shows similar large meandering than the nSR case (Figure 2) but after around 5s the single rotor splits into two and then vanishes.
(TIFF)

Table S1 Regional expression of SERCA and PLB in human myocardium (non-failing tissue, obtained from organ donors, whose hearts could not be used due to technical reasons).
(PDF)

Table S2 Percentage changes measured in ion currents in cAF as compared to nSR. I_CaL_ =  L-type calcium current, I_to_ =  transient outward K^+^ current and I_K1_ =  inward rectifier K^+^ current; I_sus_ or I_Kur_ =  sustained outward K^+^ current; ( )  =  not significant; # =  average of changes at −100 mV and −10 mV; * =  at −80 mV.
(PDF)

Table S3 Percentage changes in Ca^2+^ handling protein expression in cAF as compared to nSR. SERCA = SR Ca^2+^ ATPase; PLB =  phospholamban; SLN =  sarcolipin; and NCX = Na^+^/Ca^2+^ exchanger; ( )  =  not significant.
(PDF)

Table S4 Surface area (measured in pF) of human (right) atrial myocytes, in cAF compared to nSR.
(PDF)

Text S1 Model implementation, simulation protocols and supporting references.
(DOC)

Video S1 Color-coded transmembrane voltage distribution between 2 s and 7 s of the nSR model after the initiation of a single rotor in the tissue patch. Due to the large meandering of the wave tip, the rotor collides with the boundary of the geometry after around 7 s and vanishes.
(MP4)

Video S2 Color-coded transmembrane voltage distribution between 2 s and 10 s of the cAF model after the initiation of a single rotor in the tissue patch. The rotor is stable as the meandering of the wave tip is small.
(MP4)

Video S3 Color-coded transmembrane voltage distribution between 2 s and 6 s of the partially restored model variant (no I_K1_ and RyR remodeling, plus partial NKA block) after the initiation of a single rotor in the tissue patch. Due to the large meandering of the wave tip in combination with alternans effects also seen in Figure S12, the single rotor breaks up into two. Then, the amount of excitable tissue is too small for two rotors and after around 5.5 s the rotor vanishes.
(MP4)
