# Supplementary figures and images for: In Silico Screening of the Key Cellular Remodeling Targets in Chronic Atrial Fibrillation
Source: PLoS Comput Biol. 2014 May 22;10(5):e1003620. doi: 10.1371/journal.pcbi.1003620 (PMC4031057; doi:10.1371/journal.pcbi.1003620)

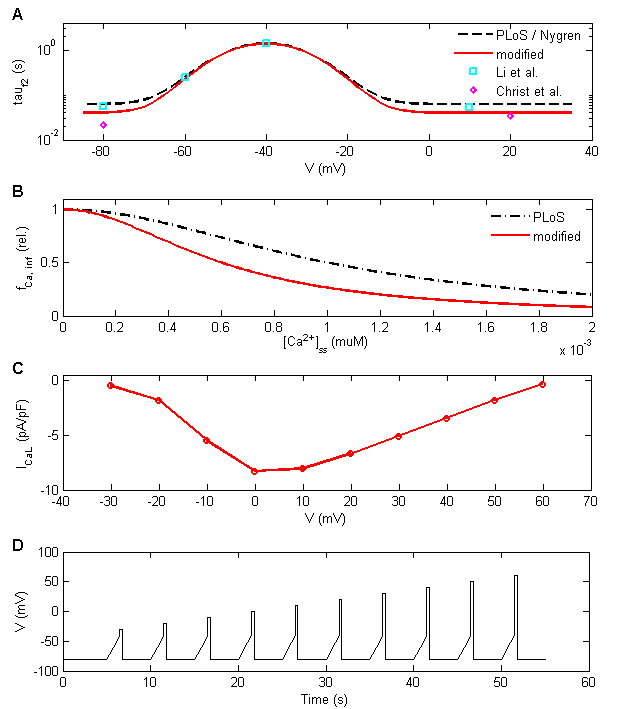

Supplement: Figure S1 — Characteristics of the modified ICaL submodel. (A) Modified time constants of inactivation and recovery, based on in vitro data of Li et al. [44] and Christ et al. [36]. (B) Modified Ca2+-dependent inactivation gate. (C) Results of an in silico voltage clamp experiment, with 10 mM EGTA. (D) Voltage clamp protocol: BCL = 5000 ms, holding potential −80 mV, 1500 ms ramp to −40 mV to inactivate INa, and pulse length of 300 ms at each testing potential. (TIF) [file pcbi.1003620.s001.tif]

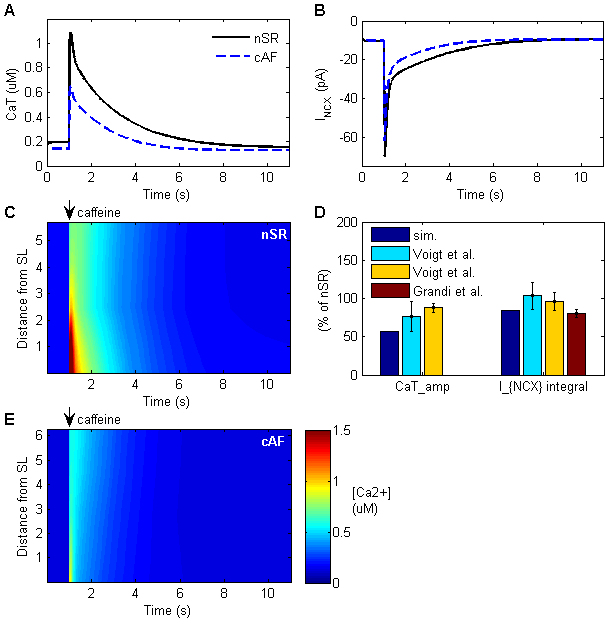

Supplement: Figure S2 — Simulated caffeine experiment. In cAF, the amplitude of the caffeine-evoked CaT (A) is decreased, in line with the in vitro results (D) of Voigt et al. [21] (cyan bar), Grandi et al. [11] and Voigt et al. [14] (yellow bar), while the integral of INCX is affected to a much smaller extent (B & D). (C & E) Spatio-temporal properties of caffeine-evoked CaT are also changed due to the cAF-remodeling of Ca2+ handling. (TIF) [file pcbi.1003620.s002.tif]

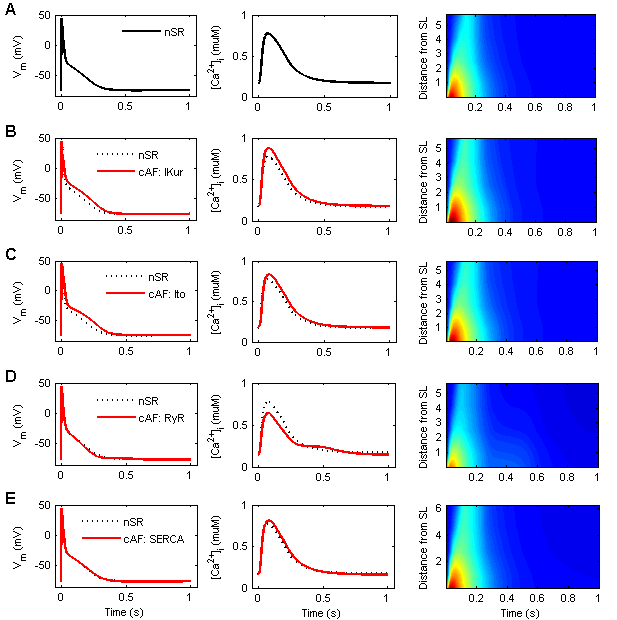

Supplement: Figure S3 — Contribution of each remodeled cellular component to changes in AP and CaT characteristics. (A) normal sinus rhythm (nSR). (B–E) four remodeled cellular components separately (IKur, Ito, RyR, SERCA), respectively. Colour scale for right column is same as in Figure 3&4 0–1.5 µM corresponds to dark blue – dark red. (TIF) [file pcbi.1003620.s003.tif]

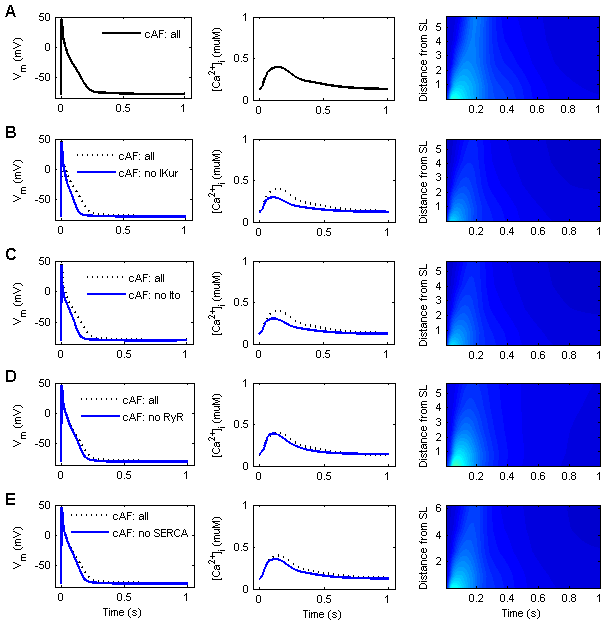

Supplement: Figure S4 — AP and CaT characteristics when a single remodeled cellular component is omitted. (A) chronic atrial fibrillation (cAF: all). (B–E) four restored cellular components (IKur, Ito, RyR, SERCA), respectively. Colour scale for right column is same as in Figure 3&4; 0–1.5 µM corresponds to dark blue – dark red. (TIF) [file pcbi.1003620.s004.tif]

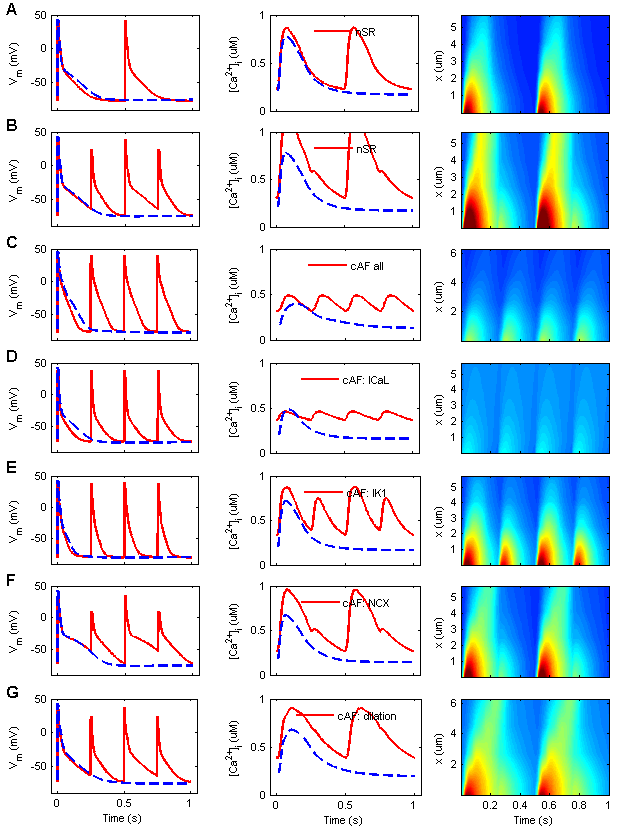

Supplement: Figure S5 — Contribution of each remodeled cellular component to changes in AP and CaT characteristics during tachy pacing (BCL = 250 ms, red solid line) as compared to normal pacing (BCL = 1000 ms, blue dashed line). (A) normal sinus rhythm (nSR) model at slower pacing (BCL = 500 ms). (B) normal sinus rhythm (nSR) model. (C) chronic atrial fibrillation (cAF: all) model. (D–G) four remodeled cellular components separately (L-type Ca2+ current, ICaL; inward rectified K+ current, IK1; Na+/Ca2+ exchanger current, INCX; and increased cell volume, dilation), respectively. Colour scale for right column is same as in Figure 3&4; 0–1.5 µM corresponds to dark blue – dark red. Model variants are identical to Figure 3. (TIF) [file pcbi.1003620.s005.tif]

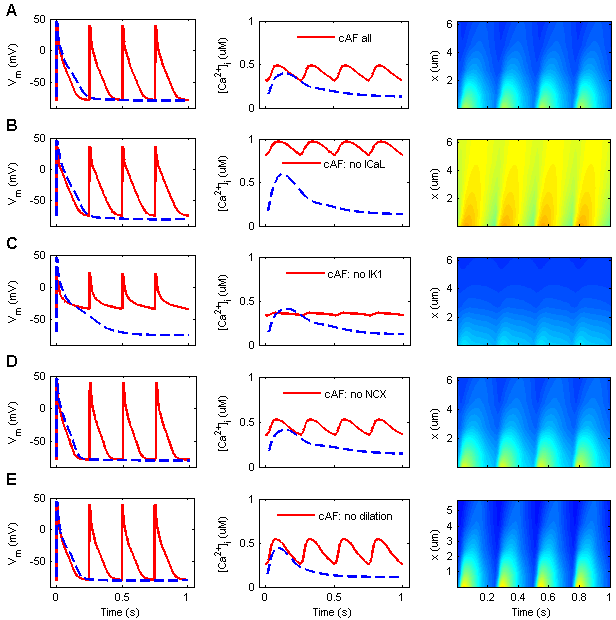

Supplement: Figure S6 — AP and CaT characteristics when a single remodeled cellular component is omitted during tachy pacing (BCL = 250 ms, red solid line) compared to normal pacing (BCL = 1000 ms, blue dashed line). (A) chronic atrial fibrillation (cAF: all). (B–E) four restored cellular components (L-type Ca2+ current, ICaL; inward rectified K+ current, IK1; Na+/Ca2+ exchanger current, INCX; and increased cell volume, dilation), respectively. Colour scale for right column is same as in Figure 3&4; 0–1.5 µM corresponds to dark blue – dark red. Model variants are identical to Figure 4. (TIF) [file pcbi.1003620.s006.tif]

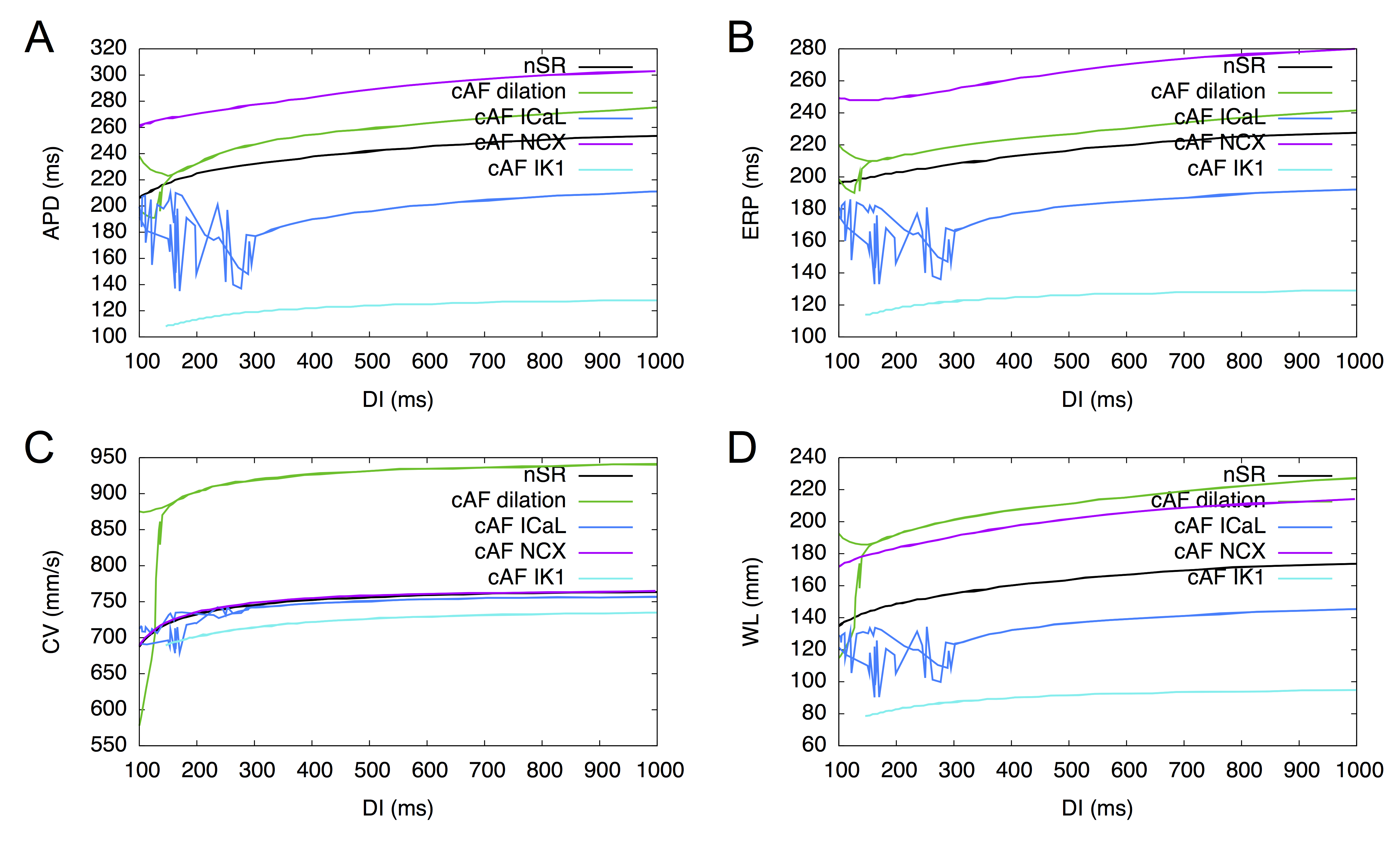

Supplement: Figure S7 — Contribution of each remodeled cellular component to normal electrophysiological properties in 1D tissue simulation. (A) APD (B) ERP, (C) CV and (D) WL. (TIFF) [file pcbi.1003620.s007.tiff]

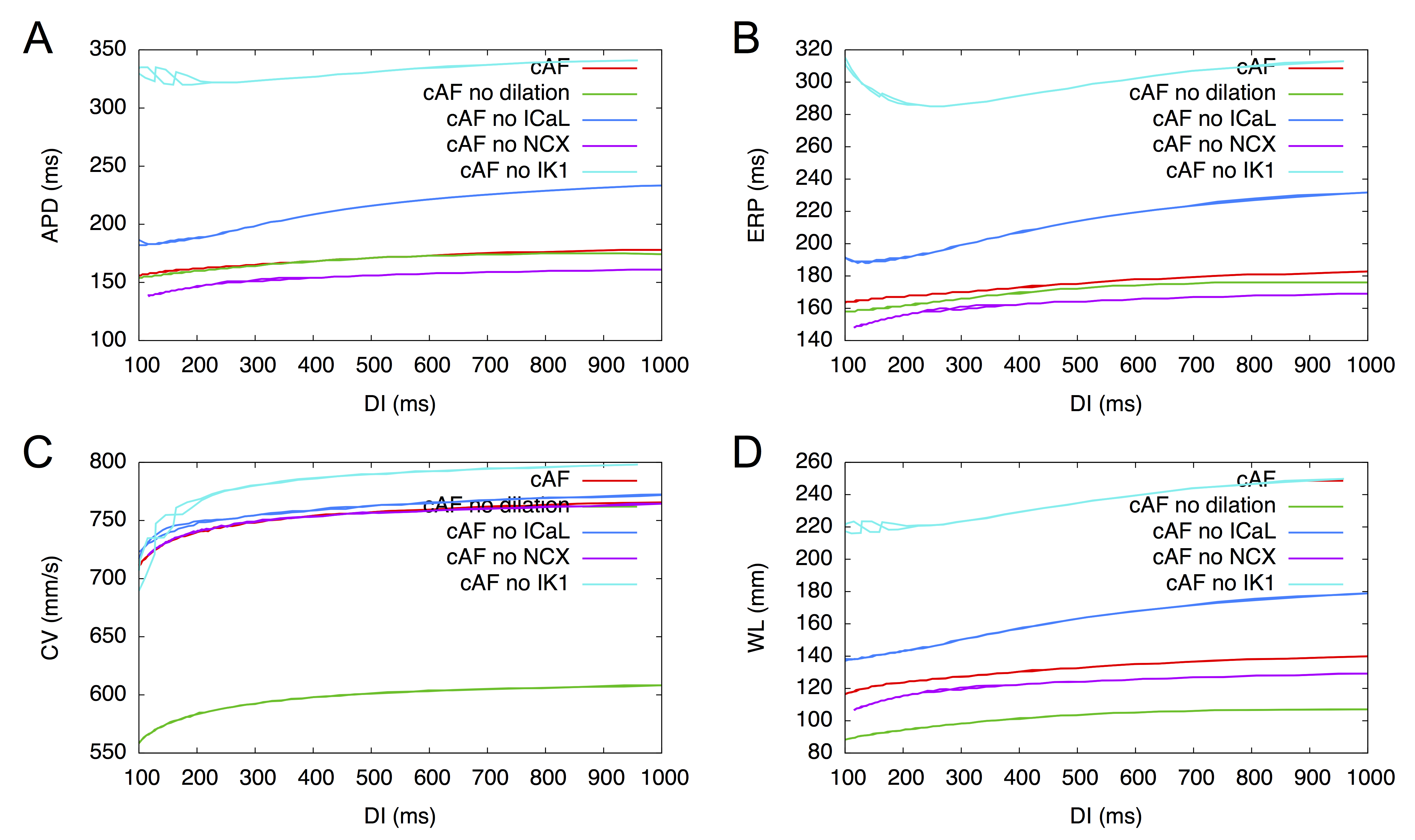

Supplement: Figure S8 — Contribution of omitting each remodeled cellular component to chronic AF electrophysiological properties in 1D tissue simulation. (A) APD (B) ERP, (C) CV and (D) WL. (TIFF) [file pcbi.1003620.s008.tiff]

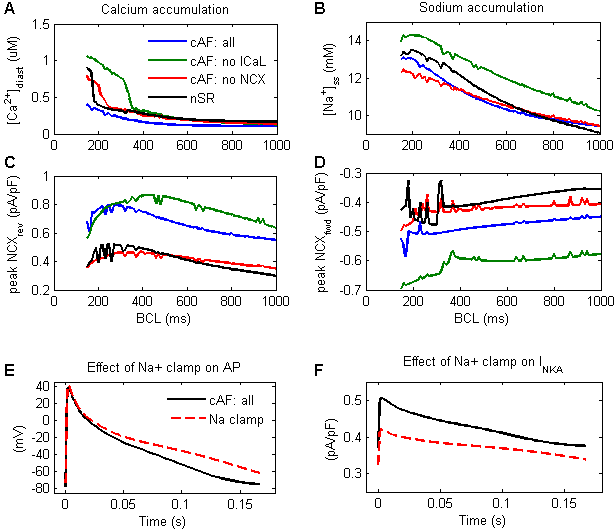

Supplement: Figure S9 — Intracellular Ca2+ and Na+ accumulation during increasingly fast pacing. (A–D) Raw data (corresponding to Figure 6 B–E), and (E–F) effect of Na+ clamp on AP and INKA. Na+ clamp, at BCL = 167 ms, was simulated by setting intracellular Na+ concentration to the value at BCL = 1000 ms. (TIF) [file pcbi.1003620.s009.tif]

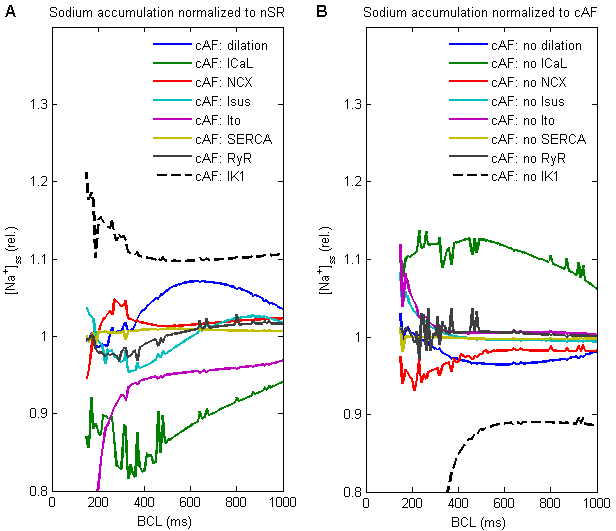

Supplement: Figure S10 — Intracellular Na+ accumulation during increasingly fast pacing for all the model variants, either including (A) or excluding (B) only a single remodeling target. (TIF) [file pcbi.1003620.s010.tif]

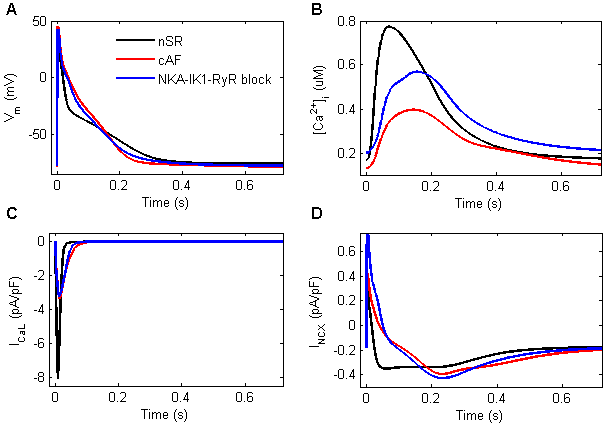

Supplement: Figure S11 — Restored RyR Ca2+ sensitivity and IK1 conductance with blocking of Na+/K+ ATPase (NKA) as an approach to increasing APD and CaTamp. (A) Faster initial and slower final phase repolarization. (B) Increased amplitude and peak CaT. (C&D) Indirect effects on ICaL and INCX. Partial block of NKA was implemented by reducing maximum current/conductance by 25%. RyR leak reduced by using the Ca2+ sensitivity and IK1 conductance, i.e. these properties were set similar to nSR. (TIF) [file pcbi.1003620.s011.tif]

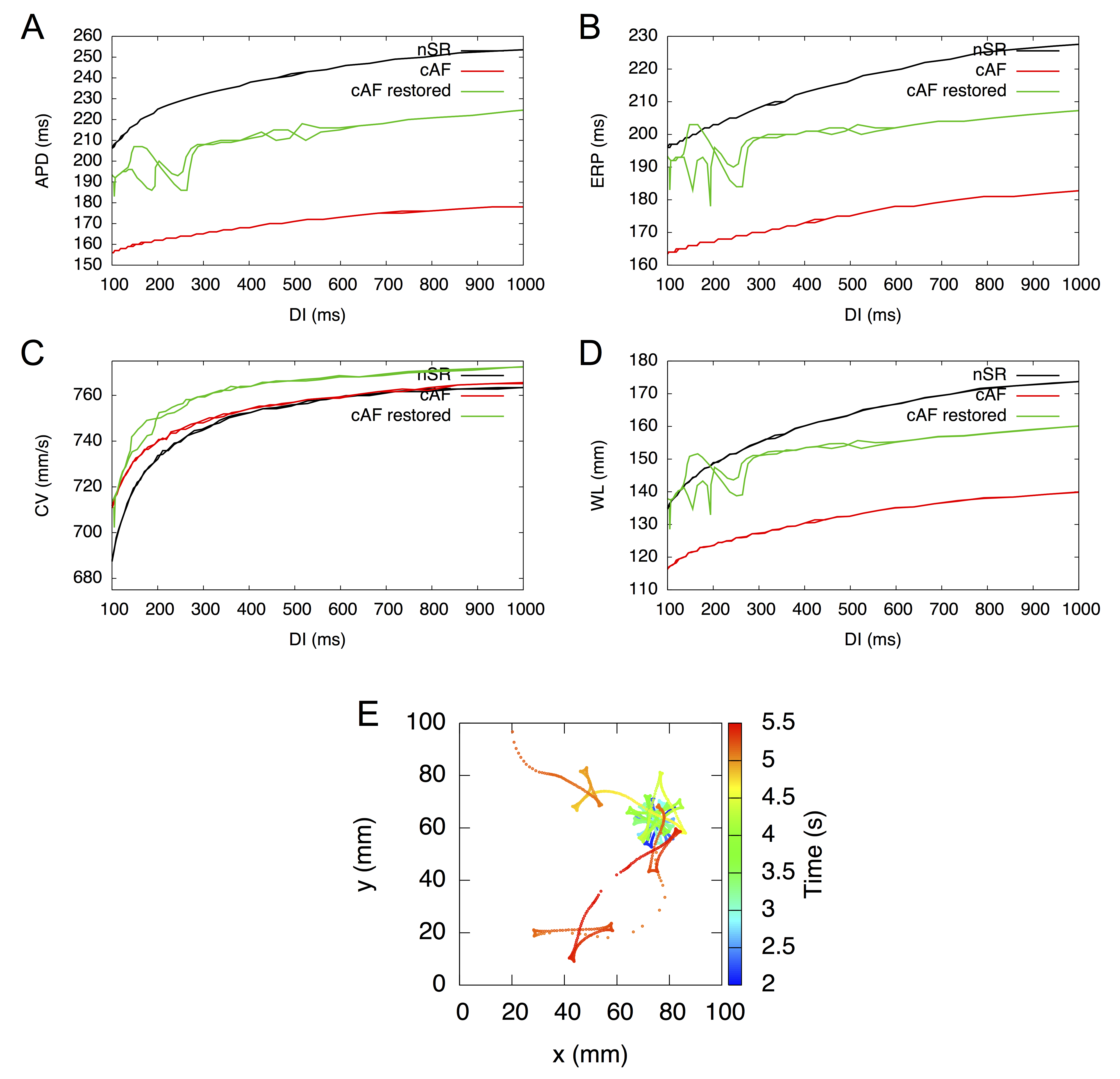

Supplement: Figure S12 — Restitution properties of the restored model variant in comparison with nSR and cAF. (A) APD (B) ERP, (C) CV and (D) WL. The restoring increases the WL almost to the level of nSR but alternans are visible for higher rates. (E) The rotor trajectory shows similar large meandering than the nSR case (Figure 2) but after around 5s the single rotor splits into two and then vanishes. (TIFF) [file pcbi.1003620.s012.tiff]
