# Supplementary material for: In Silico Screening of the Key Cellular Remodeling Targets in Chronic Atrial Fibrillation
Source: PLoS Comput Biol. 2014 May 22;10(5):e1003620. doi: 10.1371/journal.pcbi.1003620 (PMC4031057; doi:10.1371/journal.pcbi.1003620)
Supplement: Table S1 — Regional expression of SERCA and PLB in human myocardium (non-failing tissue, obtained from organ donors, whose hearts could not be used due to technical reasons). (PDF) [file pcbi.1003620.s013.pdf]

	LA/LV	LA	LV	LA/LV	Supporting Reference
SERCA	1.33				[32]
	1.70				[33]
PLB	0.525				[32]
	0.706				[33]
PLB/SERCA		1.06	2.71	0.39	[32]
		0.877	2.12	0.41	[33]
