# Supplementary material for: In Silico Screening of the Key Cellular Remodeling Targets in Chronic Atrial Fibrillation
Source: PLoS Comput Biol. 2014 May 22;10(5):e1003620. doi: 10.1371/journal.pcbi.1003620 (PMC4031057; doi:10.1371/journal.pcbi.1003620)
Supplement: Table S2 — Percentage changes measured in ion currents in cAF as compared to nSR. ICaL = L-type calcium current, Ito = transient outward K+ current and IK1 = inward rectifier K+ current; Isus or IKur = sustained outward K+ current; ( ) = not significant; # = average of changes at −100 mV and −10 mV; * = at −80 mV. (PDF) [file pcbi.1003620.s014.pdf]

Current	Change (%)	Mean (%)	Supporting Reference
I_{CaL}	-73		[34]
	-63		[35]
	-56		[36]
	-64		[37]
	-50		[38]
	-64		[39]
	-51		[40]
	-54		[24]
		**-59**	
I_{to}	-66		[41]
	-84		[34]
	-44		[42]
	-83		[43]
	-65		[37]
	-49		[44]
	-44		[19]
		**-62**	
I_{K1}	-8.6		[33]
	+102		[20]
	+106		[45] #
	+73		[46]
	+44		[37] *
	+7.2		[47]
	+75		[48]
	+96		[39]
		**+62**	
I_{sus} / I_{Kur}	-44		[41]
	(-25)		[34]
	-55		[42]
	(-24)		[43]
	-22		[44]
	-60		[19]
		**-38**	
