# Supplementary material for: In Silico Screening of the Key Cellular Remodeling Targets in Chronic Atrial Fibrillation
Source: PLoS Comput Biol. 2014 May 22;10(5):e1003620. doi: 10.1371/journal.pcbi.1003620 (PMC4031057; doi:10.1371/journal.pcbi.1003620)
Supplement: Table S3 — Percentage changes in Ca2+ handling protein expression in cAF as compared to nSR. SERCA = SR Ca2+ ATPase; PLB = phospholamban; SLN = sarcolipin; and NCX = Na+/Ca2+ exchanger; ( ) = not significant. (PDF) [file pcbi.1003620.s015.pdf]

Protein	Change (%)	Mean (%)	Supporting Reference
SERCA	-28		[49]
	(-1)		[26]
	-25		[13]
	(-17)		[25]
	(-11)		[12]
		**-16**	
PLB	+27		[49]
	(-11)		[26]
	(+28)		[13]
	(-16)		[25]
	(-19)		[12]
		**+2**	
PLB/SERCA	+57		[49]
	(-11)		[26]
	+46		[13]
	(+1)		[25]
	(-9)		[12]
		**+18**	
SLN	-47		[12]
	-47		[50]
		**-47**	
SLN/SERCA	-40		[12]
		**-40**	
NCX	(-1)		[49]
	+67		[26]
	+24		[39]
	+116		[13]
	+49		[24]
	+43		[25]
		**+50**	
