# Supplementary material for: In Silico Screening of the Key Cellular Remodeling Targets in Chronic Atrial Fibrillation
Source: PLoS Comput Biol. 2014 May 22;10(5):e1003620. doi: 10.1371/journal.pcbi.1003620 (PMC4031057; doi:10.1371/journal.pcbi.1003620)
Supplement: Table S4 — Surface area (measured in pF) of human (right) atrial myocytes, in cAF compared to nSR. (PDF) [file pcbi.1003620.s016.pdf]

nSR	cAF	cAF / nSR	Supporting Reference
72.1 ± 3.0	76.6 ± 4.8	1.06	[34]
67.6 ± 2.9	90.4 ± 8.3	1.33	[35]
92 ± 12	157 ± 14	1.71	[45]
90 ± 5	112 ± 7	1.24	[36]
84 ± 4	139 ± 11	1.65	[46]
98.3 ± 6.4	107.3 ± 5.8	1.09	[47]
88.1 ± 1.9	106.1 ± 3.8	1.20	[38]
89.4 ± 8.8	112.1 ± 9.4	1.25	[39]
78 ± 1	92 ± 2	1.18	[51]
65 ± 4.2	89 ± 5.2	1.37	[40]
70.8 ± 4.4	85.5 ± 6.1	1.21	[44]
72.3 ± 5.4	96.6 ± 7.7	1.34	[19]
