# Supplementary material for: In Silico Screening of the Key Cellular Remodeling Targets in Chronic Atrial Fibrillation
Source: PLoS Comput Biol. 2014 May 22;10(5):e1003620. doi: 10.1371/journal.pcbi.1003620 (PMC4031057; doi:10.1371/journal.pcbi.1003620)
Supplement: Text S1 — Model implementation, simulation protocols and supporting references. (DOC) [file pcbi.1003620.s017.doc]

***In silico* screening of the key cellular remodeling targets in chronic atrial fibrillation**

Jussi T. Koivumäki, Gunnar Seemann, Mary M. Maleckar, and Pasi Tavi

| **Table of Contents** |  | |  | |
| --- | --- | --- | --- | --- |
| Model Implementation |  |  | |  |
| The myocyte model: general modifications | 2 |  | |  |
| Modifications to Sarcoplasmic reticulum Ca^2+^ ATPase submodel | 2 |  | |  |
| Modifications to L-type Ca^2+^ current submodel | 3 |  | |  |
| cAF-related modifications to the cell model | 4 |  | |  |
| Simulation Protocols |  |  | |  |
| Simulations in 0D | 6 |  | |  |
| Simulations in 1D and 2D | 6 |  | |  |
| Supporting References | 8 |  | |  |

# Model Implementation

## The myocyte model: general modifications

The modeling platform of this study is our recently developed human atrial myocyte model [1] that enables the simulation of non-synchronous SR Ca^2+^ release, and the emergent spatio-temporal characteristics of intracellular Ca^2+^ dynamics: differences in time-to-peak and amplitude of junctional vs. central Ca^2+^ transient.

Conductances of I_to_ and I_Kur_ (8.25 and 2.25 nS) were corrected to be coherent with actual Maleckar *et al.* implementation, as slightly deviating parameter values were given in two different sections of the original publication [2].

## Modifications to Sarcoplasmic reticulum Ca^2+^ ATPase submodel

To enable the representation of changed expression of phospholamban (PLB) and sarcolipin (SLN) in chronic atrial fibrillation (cAF), we modified the formulation and parameters of the SERCA pump according to previously developed schemes [3,4].

In mammalian myocardium, Ca^2+^ affinity of SERCA is regulated by Phospholamban (PLB) [5]. There exist substantial regional differences in the relative expression level of PLB. For example, atrial SR has been shown to exhibit a 4-fold lower level of PLB and a 2-fold higher level of SERCA compared to ventricular SR in murine myocardium [6,7]. Human data agrees qualitatively with these findings; however the quantitative differences between atrial and ventricular protein expression are slightly smaller (Table S1). The expression of SERCA and PLB are ~1.5- and ~0.6-fold, respectively, in left atrial vs. ventricular tissue. Thus, the atrial PLB to SERCA ratio is ~0.4-fold compared to ventricular myocytes.

In addition to the regional differences in SERCA and PLB expression, recent *in vitro* findings indicate that the amino acid sequence of human PLB differs from other mammalian species (rabbit, dog, pig, mouse and rat) by amino acid 27 (lyseine vs. asparagines) [8]. Due to this mutation, the human PLB is ‘superinhibitory’ compared to other species. Accordingly, the EC50 of SERCA for Ca^2+^ was increased by ~50% (from 0.32±0.01 µM to 0.49±0.01 µM), when human PLB was expressed instead of mouse PLB in PLB knockout mice [8]. Based on a previously developed scheme [3,4],

*EC50_fwd_* = 0.15 + *EC50_fwd,PLB_* ≈ 0.30 µM

*EC50_fwd_* = 0.15 + *EC50_fwd,PLB-superinhibitory_* ≈ 0.45 µM

the findings of Zhao *et al.* would indicate that the ‘superinhibitory’ effect of human PLB is roughly twice that of other mammals. Thus, the effective PLB/SERCA ratio in human atrial myocytes is ~0.8-fold compared to ventricular myocytes of mice. This is a solid basis for comparison, since the inhibitory effect of PLB on SERCA has been extensively studied in the mouse myocardium. Accordingly, we have modified the forward and reverse affinities (µM) of SERCA as follows:

*EC50_fwd_* = 0.15 + 0.15*2*0.4 = 0.15 + 0.12 = 0.27 µM

*EC50_rev_* = 2500 – 1100*2*0.4 = 2500 – 880 = 1620 µM

In addition to PLB, SERCA function is also modulated by sarcolipin (SLN) [9,10]. In tissue from healthy human myocardium, SLN expression has been reported to be ~17.4-fold higher in left atrium vs. ventricles [11]. Based on a recent study that reported changed SLN expression in human cAF [12], we estimated the additive contribution of SLN to SERCA regulation as follows

*EC50_fwd_* = 0.15 + 0.12**PLB_ratio_* + 0.07 * SLNratio

*EC50_rev_* = 2500 – 880**PLB_ratio_* – 500 * *SLN_ratio_*

The above formulation enables also the representation of ~2-fold increase of the phosphorylation level of PLB in cAF vs. nSR [13] in line with findings that CaMK expression increases ~2-fold in cAF [14].

To compensate for the Ca^2+^ modified affinities, we decreased the total SERCA from 40 µM to 30µM, which restored the SR Ca^2+^ content to ~74 µM and the Ca^2+^ removal fraction by SERCA to 56%.

## Modifications to L-type Ca^2+^ current submodel

We reformulated the I_CaL_ equations to increase the contribution Ca^2+^- vs. voltage-dependent inactivation of the current, by removing the fast voltage-dependent inactivation gate and steepening the dependence of inactivation on sub-sarcolemmal [Ca^2+^]. Thus, the Ca^2+^- vs. voltage-dependent inactivation mechanisms correspond to the Courtemanche *et al.* formulation [15], in that fast inactivation is mediated by Ca^2+^ and slow inactivation by voltage. However, the voltage-dependent inactivation is much faster, based on a fit to in vitro data [16,17]. Recovery from inactivation was also adjusted based on the same data, and activation time constant was reduced to ¼, based on [18]. All the modification details are listed below and illustrated in Supporting Figure S1.

Steady-state activation

*I_CaL,finf_* = 0.04 + 0.96 / (1 + exp((y(i_V) + 25.5)/8.4)) + 1 / (1 + exp(-(y(i_V) - 60)/8.0))

Voltage-dependent inactivation time constant

*I_CaL,ftau_* = 1.34*exp( -((y(i_V)+40)/14.2).^2 ) + 0.04

Smaller k_Ca_ for calcium-dependent inactivation (1µM in PLoS model)

*k_CaL_* = 0.6 µM

Smaller conductance (25.3125 nS in PLoS model)

*g_CaL_* = 15 nS

## cAF-related modifications to the cell model

In summary, to simulate the effect of cellular remodeling in cAF, we accounted for

1. decreased conductances of I_CaL_ (-59%), I_to_ (-62%) and I_Kur_ (-38%), and increased conductance of I_K1_ (+62%); Table S2.
2. decreased SERCA expression (-16%), increased PLB to SERC ratio (+18%), decreased SLN to SERCA ratio (-40%), increased maximal I_NCX_ (+50%) and increased sensitivity of RyR to [Ca^2+^]_SR_ (2-fold); Table S3.
3. increased cell volume (+58%) related to cellular hypertrophy; Table S4.

*Ion currents*

Decreased conductances of I_CaL_, I_to_ and I_kur_/I_sus_ and increased conductance of I_K1_ have been reported rather consistently in cellular studies of human cAF; however the extent of changes vary quite a lot from study to study (Table S2). Our approach was to find all available data in the literature and define the cAF model based on average of these data.

Changes in activity of the rapid and slow delayed rectifiers I_Kr_ and I_Ks_ may also be involved in AF-induced electrical remodeling. Indeed, recent electrophysiological data from human atrium indicates increased I_Kr_ and I_Ks_ in cAF [19]; however, contradictory results regarding the expression of the respective channel subunits have been published, with decreased [20,21] or no change in expression of I_Kr_-encoding HERG mRNA [20], and decreased [21] or increased expression of I_Ks_ transcripts (21). Therefore, we decided to exclude these modifications from this study. When additional *in vitro* data becomes available, the model should, of course, be updated accordingly.

Recent studies have indicated a potential role for Ca^2+^-activated K^+^ current in cAF [22,23]. The reported changes in channel function are, however, contradictory. Therefore, we have, at this point, not included this aspect of cAF-related remodeling in our cell model.

*Ca^2+^ handling*

Both changes in expression and measured function of Ca^2+^ handling proteins reported in literature were included to define the model remodeled Ca^2+^ handling (Table S3). In addition, we also accounted for the effect of increased CaMKII-dependent phosphorylation of SERCA and RyR, increased ~2-fold in cAF [24,25], by decreasing the inhibitory effect of PLB and increasing Ca^2+^ sensitivity, respectively.

*Cell dilation*

The model of cell dilation in cAF is based, on reported changes in the cell membrane area, measured as picofarads, which is increased on average 1.3-fold in cAF compared to nSR (Table S4).

In addition, cell diameter has been reported to grow from 11.2 µm to 17.3 µm [26], from 14.8±0.3 µm to 17.1±0.4 µm [25] or from 12.1 µm to 18.6 µm [27], and cell length from 101.1±1.5 µm to 113.3±2.3 µm [25] in morphological studies. If we assume, based on this geometrical data, that for a cylindrical cell, length and width increase 1.1- and 1.2-fold, respectively, then the area and volume of the cell increase 1.32- and 1.58-fold, respectively. This model of cell dilation matches the average in Table S4 and is well in line with the previously reported ~40% increase in cell surface area in patients with cAF in morphological studies [28] and the 29% increase in surface area calculated based on (41). We have implemented the cell dilation in our model so that it does not change the densities of sarcolemmal ion currents.

# Simulation protocols

## Simulations in 0D

Unless stated otherwise, all simulation results were obtained with a pacing protocol, in which the virtual cell was paced starting from a quiescent steady-state with defined basic cycle lengths for 5 minutes (300 cycles) to reach a practical quasi steady-state. A basic cycle length of 1000 ms was used in all simulations except for the one presented in Figure 1 D and E, in which BCL = 2000 ms to match the *in vitro* measurement protocol for comparison.

Amplitude of stimulus current was set to 2-fold threshold of the nSR (normal sinus rhythm) model variant at BCL = 1000 ms.

The tachy pacing experiments were started from a pacing steady-state at BCL 1000 ms, and the BCL was decreased first to 500 ms and then 250 ms for 1 minute each, before calculating and displaying the variables of interest.

The CaT decay time constans was calculated by fitting a single-exponential function to the decaying phase of CaT, starting from 10% decay and ending at 90% decay.

In DAD experiments, the simulation was started from quasi steady-state at BCL = 500 ms, and a perturpation was induced at [1000, 1100] ms after the normal stimulus by either opening the subsarcolemmal RyRs (Figure 6A) or by applying a depolarising current (Figure 6C). In the nSR case, DADs were induced when the RyR open probability was increased to 0.4 or beyond, whereas increasing the open probability to 1 was not enough to induce a DAD in the other cases. When an extra current (amplitude -30.4 pA in the nSR case) was used to induce DADs, a current pulse of similar timing and carried by K^+^ ions was injected to the virtual cell.

In the dynamic APD restitution protocol, which was used to study alternans, the virtual cell was initially in a pacing steady-state at BCL = 1000 ms. A pulse train of 30 seconds was delivered at each BCL that was shortened in 10 ms steps. After each step, the pacing was either interrupted for 30 seconds to minimize the pacing memory or continued directly to include the effect of pacing memory (Ca^2+^ and Na^+^ accumulation).

## Simulations in 1D and 2D

For tissue simulation, the electrophysiological model was implemented in a modular C++ environment using a Rush–Larsen scheme for gating variables and a forward Euler scheme for the other ODEs. A time increment of 5 μs was used. Monodomain tissue simulations were performed using the parallel modular solver acCELLerate [29], wherein the finite difference method was applied. The 1D tissue strand (20 × 0.1 × 0.1 mm) and the 2D tissue patch (100 × 100 × 0.1 mm) both had cubic voxels of size 0.1 mm. The isotropic intracellular conductivity for 1D and 2D simulations was adapted to obtain a CV of ≈750mm/s at a BCL of 1s. The stimulus current amplitude was 20% above threshold.

Steady state restitution curves of APD, CV, effective refractory period (ERP) and wavelength (WL) were calculated in the 1D tissue model. For this purpose, 50 beats were calculated in single-cell first, so that models could first adapt to the different BCLs. The BCLs ranged between 0.2 and 1.3 s. Afterward, stimulation from one side of the strand initiated five consecutive beats in the tissue. The properties were than investigated following the last beat. APD_90_ was recorded three-quarters of the distance down the strand. CV was determined by dividing the distance between these measurement sites by the difference between activation times at the center of the first and the second halves of the tissue strand. The ERP was identified by applying an additional premature stimulus at the same location as a first stimulus. The time between this initial stimulus and the first premature stimulus that could initiate an AP at the center of the second half of the strand was denoted as the ERP. Furthermore, the WL, which can be defined as the distance traveled by an electrical impulse during the refractory period, was computed as the product of ERP and CV.

For 2D simulations, 50 beats were first calculated in single-cell in order to adapt the model to a BCL of 0.3 s. Three beats were then stimulated at the left side of the patch. Following the third paced beat, a premature stimulus was simulated via stimulation applied to excited tissue at the patch’s lower half. This cross-field (S1–S2) protocol was used to initiate a rotor in the patch. In case of rotor initiation success, the trajectories of the spiral cores were tracked using an algorithm based on that of Bray *et al.* [30] which identifies phase singularities. The dominant frequency was also calculated via fast Fourier transform. For this purpose, a pseudo-ECG signal as described in Seemann *et al.* [31], was computed based on the intercellular current density distribution using two electrodes at 5 mm distance from the patch and 10 mm distance between each other in the center of the patch.

# Supporting References

1. Koivumäki JT, Korhonen T, Tavi P (2011) Impact of sarcoplasmic reticulum calcium release on calcium dynamics and action potential morphology in human atrial myocytes: a computational study. PLoS Comput Biol 7: e1001067.

2. Maleckar MM, Greenstein JL, Giles WR, Trayanova NA (2009) K+ current changes account for the rate dependence of the action potential in the human atrial myocyte. AmJPhysiolHeart CircPhysiol 297: H1398–1410.

3. Shannon TR, Chu G, Kranias EG, Bers DM (2001) Phospholamban Decreases the Energetic Efficiency of the Sarcoplasmic Reticulum Ca Pump. J Biol Chem 276: 7195–7201.

4. Koivumäki JT, Takalo J, Korhonen T, Tavi P, Weckström M (2009) Modelling sarcoplasmic reticulum calcium ATPase and its regulation in cardiac myocytes. Phil Trans R Soc A 367: 2181–2202.

5. MacLennan DH, Kranias EG (2003) Phospholamban: a crucial regulator of cardiac contractility. Nat Rev Mol Cell Biol 4: 566–577.

6. Koss KL, Ponniah S, Jones WK, Grupp IL, Kranias EG (1995) Differential phospholamban gene expression in murine cardiac compartments. Molecular and physiological analyses. CircRes 77: 342–353.

7. Minajeva A, Kaasik A, Paju K, Seppet E, Lompre AM, et al. (1997) Sarcoplasmic reticulum function in determining atrioventricular contractile differences in rat heart. AmJPhysiol 273: H2498–507.

8. Zhao W, Yuan Q, Qian J, Waggoner JR, Pathak A, et al. (2006) The Presence of Lys27 Instead of Asn27 in Human Phospholamban Promotes Sarcoplasmic Reticulum Ca2+-ATPase Superinhibition and Cardiac Remodeling. Circulation 113: 995–1004.

9. Babu GJ, Bhupathy P, Timofeyev V, Petrashevskaya NN, Reiser PJ, et al. (2007) Ablation of sarcolipin enhances sarcoplasmic reticulum calcium transport and atrial contractility. ProcNatlAcadSciUSA 104: 17867–17872.

10. Bhupathy P, Babu GJ, Periasamy M (2007) Sarcolipin and phospholamban as regulators of cardiac sarcoplasmic reticulum Ca2+ ATPase. JMolCellCardiol 42: 903–911.

11. Nef HM, Möllmann H, Troidl C, Kostin S, Voss S, et al. (2009) Abnormalities in intracellular Ca2+ regulation contribute to the pathomechanism of Tako-Tsubo cardiomyopathy. Eur Heart J 30: 2155–2164.

12. Shanmugam M, Molina CE, Gao S, Severac-Bastide R, Fischmeister R, et al. (2011) Decreased sarcolipin protein expression and enhanced sarco(endo)plasmic reticulum Ca2+ uptake in human atrial fibrillation. BiochemBiophysResCommun 410: 97–101.

13. El-Armouche A, Boknik P, Eschenhagen T, Carrier L, Knaut M, et al. (2006) Molecular Determinants of Altered Ca2+ Handling in Human Chronic Atrial Fibrillation. Circulation 114: 670–680.

14. Tessier S, Karczewski P, Krause E-G, Pansard Y, Acar C, et al. (1999) Regulation of the Transient Outward K+ Current by Ca2+/Calmodulin-Dependent Protein Kinases II in Human Atrial Myocytes. CircRes 85: 810–819.

15. Courtemanche M, Ramirez RJ, Nattel S (1998) Ionic mechanisms underlying human atrial action potential properties: insights from a mathematical model. AmJPhysiolHeart CircPhysiol 275: H301–321.

16. Li GR, Nattel S (1997) Properties of human atrial ICa at physiological temperatures and relevance to action potential. AmJPhysiolHeart CircPhysiol 272: H227–235.

17. Christ T, Wüst M, Matthes J, Jänchen M, Jürgens S, et al. (2004) An aqueous extract of the marine sponge Ectyoplasia ferox stimulates L-type Ca2+-current by direct interaction with the Cav1.2 subunit. Naunyn-Schmiedeberg’s Archives of Pharmacology 370: 474–483. doi:10.1007/s00210-004-0996-4.

18. Cavalié A, McDonald TF, Pelzer D, Trautwein W (1985) Temperature-induced transitory and steady-state changes in the calcium current of guinea pig ventricular myocytes. Pflügers Archiv European Journal of Physiology 405: 294–296. doi:10.1007/BF00582574.

19. Caballero R, de la Fuente MG, Gomez R, Barana A, Amoros I, et al. (2010) In humans, chronic atrial fibrillation decreases the transient outward current and ultrarapid component of the delayed rectifier current differentially on each atria and increases the slow component of the delayed rectifier current in both. JAmCollCardiol 55: 2346–2354.

20. Brundel BJ, Van Gelder IC, Henning RH, Tuinenburg AE, Wietses M, et al. (2001) Alterations in potassium channel gene expression in atria of patients with persistent and paroxysmal atrial fibrillation: differential regulation of protein and mRNA levels for K+ channels. J Am Coll Cardiol 37: 926–932.

21. Lai LP, Su MJ, Lin JL, Lin FY, Tsai CH, et al. (1999) Changes in the mRNA levels of delayed rectifier potassium channels in human atrial fibrillation. Cardiology 92: 248–255. doi:10.1159/000006982.

22. Li M, Li T, Lei M, Tan X, Yang Y, et al. (2011) [Increased small conductance calcium-activated potassium channel (SK2 channel) current in atrial myocytes of patients with persistent atrial fibrillation]. Zhonghua Xin Xue Guan Bing Za Zhi 39: 147–151.

23. Yu T, Deng C, Wu R, Guo H, Zheng S, et al. (2012) Decreased expression of small-conductance Ca2 +-activated K+ channels SK1 and SK2 in human chronic atrial fibrillation. Life Sciences 90: 219–227. doi:10.1016/j.lfs.2011.11.008.

24. Voigt N, Trafford AW, Ravens U, Dobrev D (2009) Cellular and Molecular Determinants of Altered Atrial Ca2+ Signaling in Patients With Chronic Atrial Fibrillation. Circulation 120: S667–668.

25. Neef S, Dybkova N, Sossalla S, Ort KR, Fluschnik N, et al. (2010) CaMKII-Dependent Diastolic SR Ca2+ Leak and Elevated Diastolic Ca2+ Levels in Right Atrial Myocardium of Patients With Atrial Fibrillation. CircRes 106: 1134–1144.

26. Schotten U, Greiser M, Benke D, Buerkel K, Ehrenteidt B, et al. (2002) Atrial fibrillation-induced atrial contractile dysfunction: a tachycardiomyopathy of a different sort. Cardiovasc Res 53: 192–201.

27. Corradi D, Callegari S, Maestri R, Ferrara D, Mangieri D, et al. (2012) Differential Structural Remodeling of the Left-Atrial Posterior Wall in Patients Affected by Mitral Regurgitation with or Without Persistent Atrial Fibrillation: A Morphological and Molecular Study. Journal of Cardiovascular Electrophysiology 23: 271–279. doi:10.1111/j.1540-8167.2011.02187.x.

28. Wouters L, Guo-Shu L, Flameng W, Thijssen VLJL, Thone F, et al. (2000) Structural remodelling of atrial myocardium in patients with cardiac valve disease and atrial fibrillation. Exp Clin Cardiol 5: 158–163.

29. Seemann G, Sachse FB, Karl M, Weiss DL, Heuveline V, et al. (2010) Framework for Modular, Flexible and Efficient Solving the Cardiac Bidomain Equations Using PETSc. In: Fitt AD, Norbury J, Ockendon H, Wilson E, editors. Progress in Industrial Mathematics at ECMI 2008. Mathematics in Industry. Springer Berlin Heidelberg. pp. 363–369. Available: http://link.springer.com/chapter/10.1007/978-3-642-12110-4_55. Accessed 10 April 2013.

30. Bray M-A, Lin S-F, Aliev RR, Roth BJ, Wikswo JP (2001) Experimental and Theoretical Analysis of Phase Singularity Dynamics in Cardiac Tissue. Journal of Cardiovascular Electrophysiology 12: 716–722. doi:10.1046/j.1540-8167.2001.00716.x.

31. Seemann G, Bustamante PC, Ponto S, Wilhelms M, Scholz EP, et al. (2010) Atrial fibrillation-based electrical remodeling in a computer model of the human atrium. Computing in Cardiology, 2010. pp. 417–420.

32. Boknik P, Unkel C, Kirchhefer U, Kleideiter U, Klein-Wiele O, et al. (1999) Regional expression of phospholamban in the human heart. CardiovascRes 43: 67–76.

33. Gaborit N, Le Bouter S, Szuts V, Varro A, Escande D, et al. (2007) Regional and tissue specific transcript signatures of ion channel genes in the non-diseased human heart. JPhysiol 582: 675–693.

34. Bosch RF, Zeng X, Grammer JB, Popovic K, Mewis C, et al. (1999) Ionic mechanisms of electrical remodeling in human atrial fibrillation. Cardiovasc Res 44: 121–131.

35. Van Wagoner DR, Pond AL, Lamorgese M, Rossie SS, McCarthy PM, et al. (1999) Atrial L-Type Ca2+ Currents and Human Atrial Fibrillation. CircRes 85: 428–436.

36. Skasa M, Jungling E, Picht E, Schondube F, Luckhoff A (2001) L-type calcium currents in atrial myocytes from patients with persistent and non-persistent atrial fibrillation. Basic ResCardiol 96: 151–159. doi:10.1007/s003950170065.

37. Workman AJ, Kane KA, Rankin AC (2001) The contribution of ionic currents to changes in refractoriness of human atrial myocytes associated with chronic atrial fibrillation. CardiovascRes 52: 226–235.

38. Christ T, Boknik P, Wohrl S, Wettwer E, Graf EM, et al. (2004) L-type Ca2+ current downregulation in chronic human atrial fibrillation is associated with increased activity of protein phosphatases. Circulation 110: 2651–2657.

39. Gaborit N, Steenman M, Lamirault G, Le Meur N, Le Bouter S, et al. (2005) Human Atrial Ion Channel and Transporter Subunit Gene-Expression Remodeling Associated With Valvular Heart Disease and Atrial Fibrillation. Circulation 112: 471–481.

40. Greiser M, Halaszovich CR, Frechen D, Boknik P, Ravens U, et al. (2007) Pharmacological evidence for altered src kinase regulation of ICa,L in patients with chronic atrial fibrillation. Naunyn Schmiedebergs ArchPharmacol 375: 383–392.

41. Van Wagoner DR, Pond AL, McCarthy PM, Trimmer JS, Nerbonne JM (1997) Outward K+ Current Densities and Kv1.5 Expression Are Reduced in Chronic Human Atrial Fibrillation. CircRes 80: 772–781.

42. Brandt MC, Priebe L, Böhle T, Südkamp M, Beuckelmann DJ (2000) The Ultrarapid and the Transient Outward K+Current in Human Atrial Fibrillation. Their Possible Role in Postoperative Atrial Fibrillation. JMolCellCardiol 32: 1885–1896.

43. Grammer JB, Bosch RF, Kuhlkamp V, Seipel L (2000) Molecular remodeling of Kv4.3 potassium channels in human atrial fibrillation. JCardiovascElectrophysiol 11: 626–633.

44. Christ T, Wettwer E, Voigt N, Hala O, Radicke S, et al. (2008) Pathology-specific effects of the IKur/Ito/IK,ACh blocker AVE0118 on ion channels in human chronic atrial fibrillation. Br J Pharmacol 154: 1619–1630.

45. Dobrev D, Graf E, Wettwer E, Himmel HM, Hala O, et al. (2001) Molecular Basis of Downregulation of G-Protein-Coupled Inward Rectifying K+ Current (IK,ACh) in Chronic Human Atrial Fibrillation: Decrease in GIRK4 mRNA Correlates With Reduced IK,ACh and Muscarinic Receptor-Mediated Shortening of Action Potentials. Circulation 104: 2551–2557.

46. Dobrev D, Wettwer E, Kortner A, Knaut M, Schüler S, et al. (2002) Human inward rectifier potassium channels in chronic and postoperative atrial fibrillation. Cardiovasc Res 54: 397–404.

47. Balana B, Dobrev D, Wettwer E, Christ T, Knaut M, et al. (2003) Decreased ATP-sensitive K(+) current density during chronic human atrial fibrillation. JMolCellCardiol 35: 1399–1405.

48. Dobrev D, Friedrich A, Voigt N, Jost N, Wettwer E, et al. (2005) The G Protein-Gated Potassium Current IK,ACh Is Constitutively Active in Patients With Chronic Atrial Fibrillation. Circulation 112: 3697–3706.

49. Brundel BJJM, Van Gelder IC, Henning RH, Tuinenburg AE, Deelman LE, et al. (1999) Gene expression of proteins influencing the calcium homeostasis in patients with persistent and paroxysmal atrial fibrillation. Cardiovasc Res 42: 443–454.

50. Uemura N, Ohkusa T, Hamano K, Nakagome M, Hori H, et al. (2004) Down-regulation of sarcolipin mRNA expression in chronic atrial fibrillation. EurJClinInvest 34: 723–730.

51. Pau D, Workman AJ, Kane KA, Rankin AC (2007) Electrophysiological and arrhythmogenic effects of 5-hydroxytryptamine on human atrial cells are reduced in atrial fibrillation. Journal of Molecular and Cellular Cardiology 42: 54–62. doi:10.1016/j.yjmcc.2006.08.007.

52. Grandi E, Pandit SV, Voigt N, Workman AJ, Dobrev D, et al. (2011) Human Atrial Action Potential and Ca2+ Model: Sinus Rhythm and Chronic Atrial Fibrillation. Circ Res 109: 1055–1066.

53. Voigt N, Li N, Wang Q, Wang W, Trafford AW, et al. (2012) Enhanced Sarcoplasmic Reticulum Ca2+ Leak and Increased Na+-Ca2+ Exchanger Function Underlie Delayed Afterdepolarizations in Patients With Chronic Atrial Fibrillation. Circulation 125: 2059–2070. doi:10.1161/CIRCULATIONAHA.111.067306.
